# Loss of chromosome 9p21 is associated with a poor prognosis in adenosquamous carcinoma of the pancreas

**DOI:** 10.1093/pcmedi/pbad030

**Published:** 2023-11-07

**Authors:** Yina Jiang, Yinying Wu, Liwen Zhang, Yan Wang, Guiping Xu, Yuan Deng, Liang Han, Enxiao Li, Qingyong Ma, Mian Xu, Zheng Wu, Zheng Wang

**Affiliations:** Department of Pathology, The First Affiliated Hospital of Xi'an Jiaotong University, Xi'an 70061, China; Pancreatic disease treatment center, Xi'an Jiaotong University, Xi'an 70061, China; Department of Medical Oncology, The First Affiliated Hospital of Xi'an Jiaotong University, Xi'an 70061, China; Pancreatic disease treatment center, Xi'an Jiaotong University, Xi'an 70061, China; OrigiMed Co. Ltd, Shanghai 201114, China; OrigiMed Co. Ltd, Shanghai 201114, China; Department of Radiology, The First Affiliated Hospital of Xi'an Jiaotong University, Xi'an 70061, China; Pancreatic disease treatment center, Xi'an Jiaotong University, Xi'an 70061, China; Department of Pathology, The First Affiliated Hospital of Xi'an Jiaotong University, Xi'an 70061, China; Pancreatic disease treatment center, Xi'an Jiaotong University, Xi'an 70061, China; Department of Hepatobiliary Surgery, The First Affiliated Hospital of Xi'an Jiaotong University, Xi'an 70061, China; Pancreatic disease treatment center, Xi'an Jiaotong University, Xi'an 70061, China; Department of Medical Oncology, The First Affiliated Hospital of Xi'an Jiaotong University, Xi'an 70061, China; Pancreatic disease treatment center, Xi'an Jiaotong University, Xi'an 70061, China; Department of Hepatobiliary Surgery, The First Affiliated Hospital of Xi'an Jiaotong University, Xi'an 70061, China; Pancreatic disease treatment center, Xi'an Jiaotong University, Xi'an 70061, China; OrigiMed Co. Ltd, Shanghai 201114, China; Department of Hepatobiliary Surgery, The First Affiliated Hospital of Xi'an Jiaotong University, Xi'an 70061, China; Pancreatic disease treatment center, Xi'an Jiaotong University, Xi'an 70061, China; Department of Hepatobiliary Surgery, The First Affiliated Hospital of Xi'an Jiaotong University, Xi'an 70061, China; Pancreatic disease treatment center, Xi'an Jiaotong University, Xi'an 70061, China

**Keywords:** adenosquamous carcinoma of the pancreas, genomic pattern, 9p21 loss, biomarker, prognostic

## Abstract

Adenosquamous carcinoma of the pancreas (ASCP) is a rare histological subtype of pancreatic cancer with a poor prognosis and a high metastasis rate. However, little is known about its genomic landscape and prognostic biomarkers. A total of 48 ASCP specimens and 98 pancreatic ductal adenocarcinoma (PDAC) tumour specimens were sequenced to explore the genomic landscape and prognostic biomarkers. The homozygous deletion of the 9p21.3 region (including *CDKN2A, CDKN2B*, and *MTAP)* (9p21 loss) occurred in both ASCP and PDAC, and a higher frequency of 9p21 loss was observed in ASCP (12.5% vs 2.0%, *P* = 0.022). Notably, 9p21 loss was significantly associated with poor disease-free survival (DFS) in ASCP patients (mDFS (Median DFS) = 4.17 vs 7.33 months, HR (Hazard Ratio) = 3.70, *P* = 0.009). The most common gene alterations in patients with ASCP were *KRAS* (96%), *TP53* (81%), *CDKN2A* (42%), *SMAD4* (21%), *CDKN2B* (13%), and *FAT3* (13%). The mutation rates of *ACVR2A* (6.25% vs 0%), *FANCA* (6.25% vs 0%), *RBM10* (6.25% vs 0%), and *SPTA1* (8.33% vs 1.02%) were significantly higher in ASCP than in PDAC. In conclusion, we have comprehensively described the genomic landscape of the largest cohort of ASCP patients to date and highlight that 9p21 loss may be a promising prognostic biomarker for ASCP, which provides a molecular basis for prognosis prediction and new therapeutic strategies for ASCP.

## Introduction

Adenosquamous carcinoma of the pancreas (ASCP), a rare histological subtype of pancreatic cancer,^1^, ^2^ constitutes ∼1–4% of all pancreatic cancers.^3^, ^4^ According to the World Health Organization classification of pancreatic cancers, ASCP is characterized by a variable proportion of adenocarcinoma and squamous carcinoma components, accounting for at least 30% of tumour cells. Similar to pancreatic ductal adenocarcinoma (PDAC), surgical excision is the only radical cure for patients.^5^ Since ASCP generally occurs in the body of the pancreas and the tumours are usually larger than those of PDAC,^6^ surgical resection is more feasible for ASCP, with an R0 resection rate of 58%–67%.^2^, ^7^ However, ASCP tends to be more aggressive and has a poorer prognosis than PDAC. Moreover, it has a higher metastasis rate with unfavourable clinical outcomes.^4^, ^6^ Therefore, finding new therapeutic strategies to improve the prognosis and survival of patients with ASCP is essential.

To better understand the molecular pathology of ASCP, researchers have applied next-generation sequencing (NGS) to examine the genomic landscape. These examinations have increased our understanding of the molecular basis of ASCP. Fang *et al*. investigated 17 ASCP specimens using whole-exome sequencing and determined that *KRAS, TP53*, and *SMAD4* are the most frequently mutated genes. Multiple 3p regions in ASCP showed significant copy number losses compared with those in PDAC.^8^ Due to the rarity of this subtype, most of the available studies have been limited by small sample sizes. Moreover, since it has a poorer prognosis than PDAC, it is urgent to seek optimal treatments, including chemotherapy, radiotherapy, targeted therapy, and immunotherapy. The scarcity of tumour samples suitable for high-throughput sequencing analyses has hindered genomic studies of this fatal subtype. Thus, information about the genomic landscape, potential oncogenic driver mutations, prognosis-related gene alterations of ASCP, and their relationship with PDAC is limited.

In the present study, we investigated the genomic landscape and prognostic biomarkers in ASCP. Understanding the underlying factors contributing to poor survival might be beneficial to the treatment of ASCP. We also compared ASCP and PDAC data to explore the molecular differences between these two subtypes.

## Materials and methods

### Ethics approval and consent to participate

Ethics approval was granted by the Ethics Committee of the First Affiliated Hospital of Xi'an Jiaotong University, based on the World Medical Association Declaration of Helsinki (No. XJTU1AF2021LSK-053).

### Patients and tumour samples

Written informed consent was obtained from all participants or their legal guardian/next of kin, allowing for the collection and use of their tumor samples. Tumour samples from 48 patients with ASCP and 98 patients with PDAC were collected at the First Affiliated Hospital of Xi'an Jiaotong University from 2012 to 2020. Pathological diagnoses of ASCP were confirmed according to the 5^th^ edition of the World Health Organization Classification of Tumours and ensured that both adenocarcinoma and squamous carcinoma components were contained in each sample. Pathological sections were cut from formalin-fixed paraffin-embedded (FFPE) tumour blocks for subsequent use. Both tumour tissue and matched normal blood samples were collected from each patient. Before sequencing, the samples were reviewed by two experienced pathologists who evaluated the FFPE tumour samples for tumour cell percentages in the sequencing laboratory. Complete medical records included patient age, gender, pathological reports, operation time, surgical approach, and survival information.

We also downloaded copy-number alterations (CNA) data for 183 pancreatic adenocarcinoma (PAAD) samples, along with corresponding clinical information, gene expression and mutation data, from the cBioPortal website (https://www.cbioportal.org/).

### Sequencing data analysis and tumour mutational burden

Genomic mutations were identified using an NGS-based YuanSu™ (OrigiMed, Shanghai, China, a College of American Pathologists-accredited and Clinical Laboratory Improvement Amendments-certified laboratory) gene panel, covering all coding exons of 450 cancer-related genes in solid tumours. At least 50  ng of cancer tissue DNA was extracted from each 40 mm FFPE tumour sample using a DNA extraction kit (QIAamp DNA FFPE Tissue Kit; Qiagen, Hilden, Germany) according to the manufacturer's protocols. Genomic alterations (GAs) were identified using the procedure described by Cao *et al*.: single nucleotide variants (SNVs) were identified using MuTect v1.7, and insertion deletions (indels) were identified using PINDELV0.2.5.^9^ According to the ExAC, 1000 Genomes, dbSNP build 155, and ESP6500SI-V2 databases, variants with population frequencies >0.1% were grouped as single-nucleotide polymorphisms (SNPs) and excluded from further analysis. The remaining variants were annotated using ANNOVAR and SnpEff v.3.0. Copy number variations (CNVs) were identified using Control-FREEC (v9.7) with the following parameters: window = 50 000 and step = 10 000. Gene fusions were detected using an in-house pipeline. Gene rearrangements were assessed using Integrative Genomics Viewer.^10^ Our sequencing data were compared with those of The Cancer Genome Atlas (TCGA) database to eliminate bias, and it was found that the mutation frequency of our sequencing data was basically the same as that of the TCGA-PAAD cohort (Fig. S1, see online supplementary material).

Tumour mutational burden (TMB) was estimated by counting coding somatic mutations, including SNVs and indels, per megabase of the sequence examined for each patient. As cut-offs for categorizing the TMB status of ASCP have not been defined, we used the criteria established in a previous study for other types of tumours.^11^ In this study, the median TMB was 2.75 mutations/megabase (muts/Mb); TMB-L was defined as <10 muts/Mb and TMB-H as ≥10 muts/Mb of sequenced DNA.

### Differential expression and functional enrichment analyses

In the TCGA-PAAD cohort, the Wilcoxon test was utilized to identify differentially expressed genes between the 9p21 loss and 9p21 wild type (WT) groups, and the results were visualized in a volcano plot. The Gene Ontology (GO) annotation and Kyoto Encyclopedia of Genes and Genomes (KEGG) pathway analysis were performed with DAVID 6.8 bioinformatics resources and plotted by using the ggplot2 package in R. Lists of genes involved in various signalling pathways were acquired from the KEGG database (http://www.genome.jp/kegg/pathway.html) and are provided in Table S1, see online supplementary material. Gene set enrichment analysis (GSEA) was performed using GSEA software (version 4.1.0). To evaluate the tumour immune microenvironment, single-sample GSEA was carried out by using the “GSVA” R package.

### Immunohistochemistry

Immunohistochemical (IHC) staining of FFPE tissues was performed using anti-programmed death-ligand 1 (PD-L1) antibodies (clone 22C3, Cat. M3653, DAKO). Dilutions (22C3; 1 : 50) of the primary antibodies were used for antigen detection. All slides were counterstained with hematoxylin. The PD-L1 level was reported as the combined positive score (CPS), which was defined as the number of PD-L1-positive cells divided by the total tumour cells, multiplied by 100. CPS < 1 was defined as PD-L1 negative; 1 ≤ CPS < 10 was defined as PD-L1 with low expression; and CPS ≥ 10 was defined as PD-L1 with high expression. Genes were captured and sequenced, with a mean depth of 800×, using Illumina NextSeq 500 (Illumina, CA, USA) as described by Frampton *et al*.^12^ IHC for p16 status was also conducted using anti-human p16 mouse monoclonal antibody (clone MX007) in tissues from patients with 9p21 loss and 9p21 WT.

### Statistical analyses

All analyses were performed using R software version v 4.0.3. Fisher's exact test was used for the association analysis of categorical variables. Student's *t* test and the Wilcoxon rank-sum test were used for the association analysis of normally and non-normally distributed data, respectively. The Kruskal‒Wallis test was used to analyse the association between groups of non-parametric data. A Cox proportional hazards regression model was used to analyse the relationship between predictor variables and the time to an event, such as overall survival (OS) or disease-free survival (DFS). Statistical significance was set at *P* < 0.05.

## Results

### Patient characteristics

A total of 48 patients with ASCP and 98 patients with PDAC were recruited for this study. The clinical characteristics of the patients are provided in Table 1. Of 48 patients, 28 (58.3%) were male and 20 (41.7%) were female. The mean age of the patients was 58 years (ranging from 33 to 83 years). The median TMB was 2.75 muts/Mb (ranging from 0.5 to 21.7 muts/Mb), whereas a TMB value >10 muts/Mb was detected in only one patient. The ratio of males to females among the 98 patients with PDAC was ∼1 : 1, distal metastasis was the most common metastasis type, and tumour stages II and IV were most common. Detailed information on the prognosis and treatment of ASCP is shown in Table S2, see online supplementary material.

**Table 1.** Clinicopathological characteristics of patients with ASCP and PDAC.

Variable	ASCP (*n* = 48)	PDAC (*n* = 98)
**Age (years), mean (range)**	58 (33–83)	59 (35–84)
**TMB, mean (range)**	2.75 (0.5–21.7)	3 (0–47)
**Gender**		
Female	20 (41.7%)	40 (40.8%)
Male	28 (58.3%)	58 (59.2%)
**Tumour stage**		
I	13 (27.1%)	13 (13.3%)
II	16 (33.3%)	28 (28.5%)
III	7 (14.6%)	14 (14.3%)
IV	11 (22.9%)	32 (32.7%)
Undefined	1 (2.1%)	11 (11.2%)
**Tumour differentiation**		
High	1 (2.1%)	2 (2.0%)
Moderate	26 (54.2%)	59 (60.2%)
Low	12 (25%)	17 (17.3%)
Undefined	9 (18.7%)	20 (20.3%)
**Metastasis**		
Lymph node	14 (29.2%)	27 (27.6%)
Vascular invasion	9 (18.2%)	12 (12.2%)
Nerve invasion	15 (31.3%)	37 (37.8%)
Distal metastasis	15 (31.3)	51 (52.0%)
**CA19-9**		
≤37	4 (8.3)	15 (15.3%)
37–100(≤)	4 (8.3)	5 (5.1%)
100–1000(≤)	14 (29.2)	37 (37.8%)
>1000	11 (22.9)	13 (13.3%)
Undefined	15 (31.3)	28 (28.5%)
**Family history of cancer**		
Yes	12 (25)	23 (23.5%)
No	33 (68.7)	62 (63.2%)
Undefined	3 (6.3)	13 (13.3%)

### Histological pathology of ASCP

**Figure 1.**
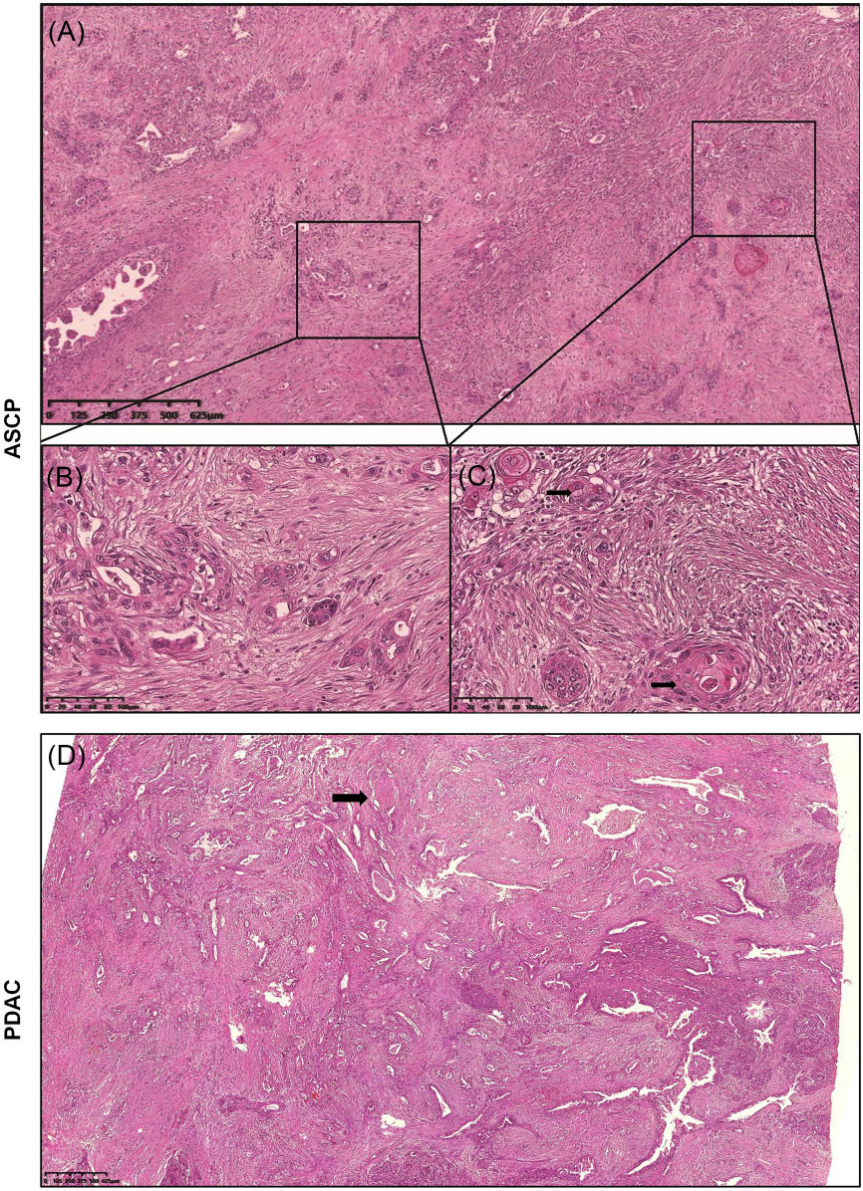
Histopathological features of ASCP (hematoxylin and eosin staining). (**A**) Irregular glandular structures and sheets of tumours were observed in the fibrous stroma. (**B**) In the adenocarcinoma component, atypical cells formed incomplete or complex glands. The nuclear-to-cytoplasmic ratio was relatively high, and nucleolar staining was significant. (**C**) The squamous cell carcinoma components demonstrated typical keratin pearls and single-cell keratinization. The polygonal tumour cells displayed abundant eosinophilic cytoplasm and distinct borders. (**D**) Histopathological features of PDAC (hematoxylin and eosin staining).

ASCP is defined as a lesion containing both adenocarcinoma and squamous carcinoma within the same tumour (Fig. 1A–C). Adenocarcinoma forms incomplete or complex glands, with a high nuclear-to-cytoplasmic ratio and prominent nucleoli. The squamous component is characterized by sheets of polygonal cells with dense eosinophilic cytoplasm, distinct cell borders, varying degrees of keratinization, typical keratin pearls, and single-cell keratinization. The pathology characteristics of PDAC were similar to those of the adenocarcinoma components in ASCP (Fig. 1D).

### Genetic profiling of ASCP and TMB

**Figure 2.**
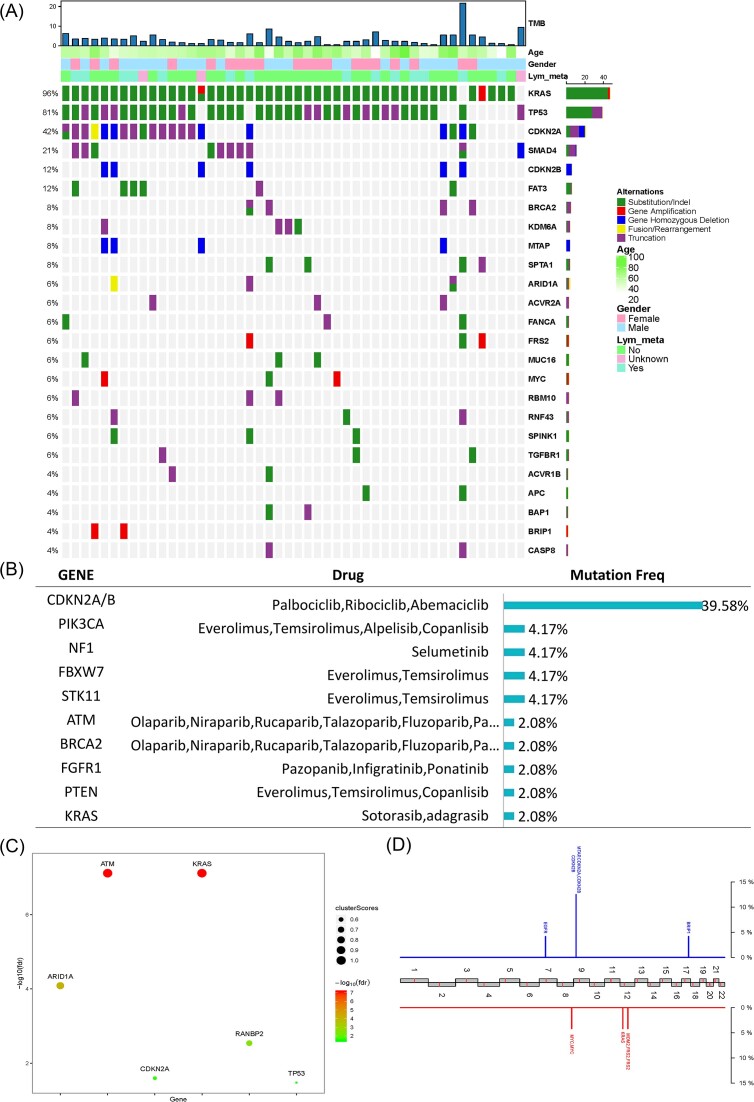
Genomic landscape of 48 ASCP patients. (**A**) The features of GAs. The panel shows the matrix of mutations coloured according to the type of mutation. Each column denotes an individual patient and each row represents a gene. (**B**) The distributions indicate GAs. (**C**) Onco-driver genes represented in the ASCP samples. (**D**) Recurrent copy number variations. Red represents copy number gains and blue represents losses.

Tumour samples from the 48 patients with ASCP were sequenced using NGS. Genetic profiling is shown in Fig. 2A. A total of 360 variations in 147 genes, including 200 (55.6%) substitutions/indels, 56 (15.6%) gene amplifications, 77 (21.4%) truncations, 7 (1.9%) fusions/rearrangements, and 20 (5.6%) gene homozygous deletions, were detected in 48 ASCP patients. The landscape of the genetic alterations was mapped. The most common genomic alterations (GAs) among the 48 ASCP patients included *KRAS* (96%), *TP53* (81%), *CDKN2A* (42%), *SMAD4* (21%), *CDKN2B* (13%), and *FAT3* (13%) (Fig. 2A). Additionally, 48% (23/48) of patients harboured potential drug-related GAs. The potential actionable GAs included *CDKN2A/B* (39.58%), phosphatidylinositol-4,5-bisphosphate 3-kinase catalytic subunit alpha (*PIK3CA*) (4.17%), *NF1* (4.17%), *FBXW7* (4.17%), *STK11* (4.17%), *ATM* (2.08%), *BRCA2* (2.08%), *FGFR1* (2.08%), phosphatase and tensin homolog (*PTEN*) (2.08%), and *KRAS* (2.08%) (Fig. 2B). The oncodriver genes *KRAS, ATM, ARID1A, RANBP2, CDKN2A*, and *TP53* were also identified (Fig. 2C). Recurrent CNVs were investigated using NGS-based copy number profiling. Losses of 7p (*EGFR*), 9p (*MTAP, CDKN2A*, and *CDKN2B*), and 17q (*BRIP1*), as well as gains in 8q (*MYC*), 12p (*KRAS*), and 12q (*MDM2* and *FRS2*), were detected (Fig. 2D). Furthermore, TMB was significantly associated with *ACVR2A, BRCA2, CASP8, CDKN2A, EPHB1, FRS2, SPTA1, KRAS*, and *STK11* mutations. Mutations in *ACVR2A, BRCA2, CASP8, CDKN2A, EPHB1, FRS2*, and *SPTA1* may lead to a significant increase in TMB, while mutations in *KRAS* and *STK11* may lead to a significant decrease in TMB (Fig. S2A, see online supplementary material).

To further understand the molecular characteristics of ASCP, gene mutation-related pathways of ASCP were analysed. We analysed the regulatory pathways involving mutated genes to understand the contribution of these mutations to selective advantages during tumorigenesis. KEGG pathway analysis showed that the mutated genes were mainly involved in the Ras, phosphoinositide 3-kinase (PI3K)-Akt, and cell cycle signalling pathways. Notably, many genes were related to platinum resistance, suggesting that patients carrying related mutations should be cautious about using platinum drugs. GO analysis showed that the mutated genes mainly occur in biological processes (BP), especially in the positive regulation of cell proliferation (Fig. S2B). Additionally, the correlation between the related signalling pathways and TMB was explored. Our results showed that mutations in homologous recombination (HR)- and cell cycle pathway-associated genes were significantly correlated with an increase in TMB (*P* < 0.01) (Fig. S2C, D).

### Comparison of genetic profiling between ASCP and PDAC

**Figure 3.**
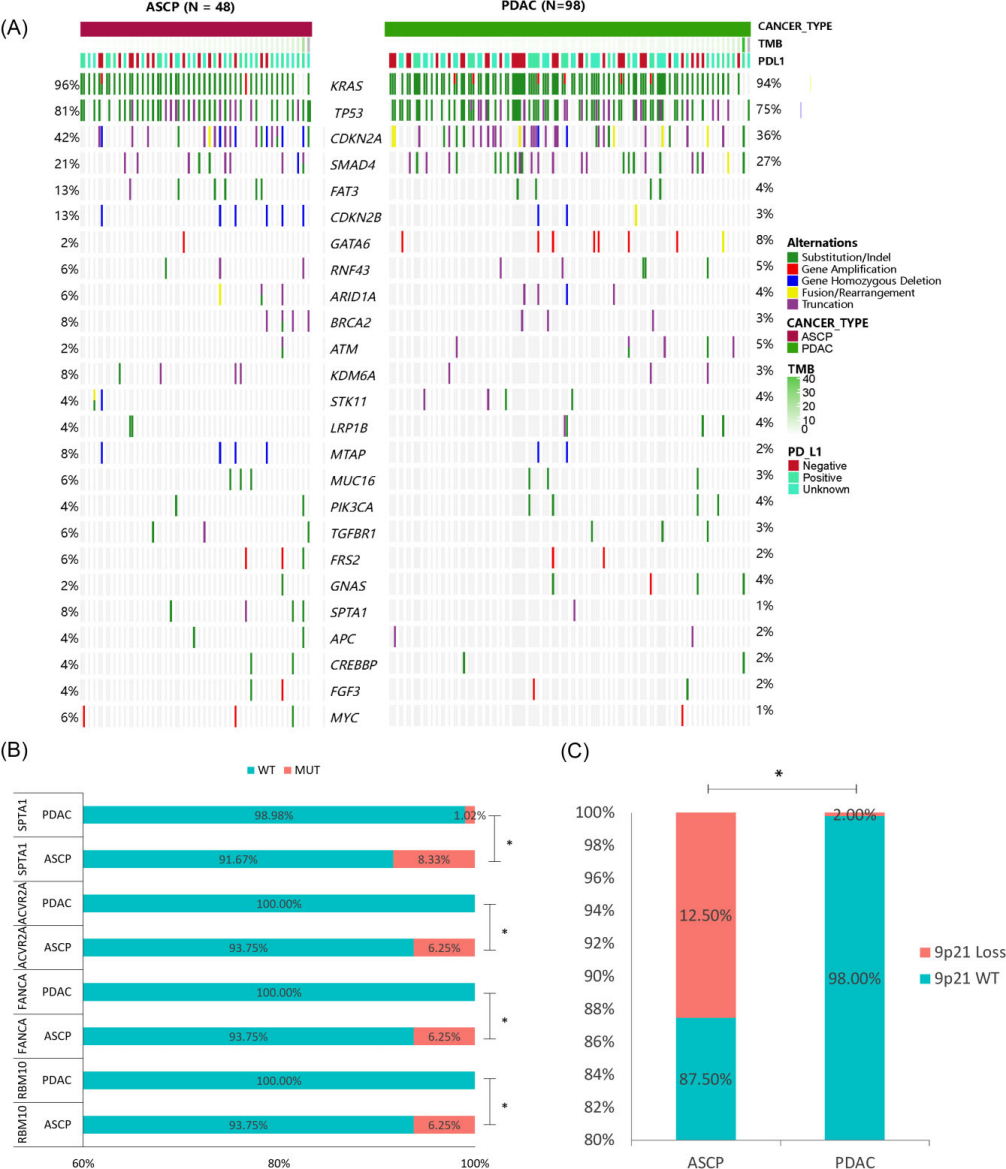
Comparison of genetic characterization between ASCP and PDAC. (**A**) The profiles of GAs. Dark red represents 48 ASCP patients and green represents 98 PDAC patients. The panel shows the matrix of mutations coloured according to the type of mutation. Green: substitution/indel; red: gene amplification; blue: gene homozygous deletion; yellow: fusion/rearrangement; purple: truncation. (**B**) Genes with a significant difference in mutation frequency between ASCP and PDAC. (**C**) Comparison of the 9p21 homologous deletion ratio in ASCP and PDAC. *P<0.05.

To better investigate the molecular differences between ASCP and PDAC, we analysed the mutational characteristics. The most common genetic alterations in 48 ASCP patients included *KRAS* (96%), *TP53* (81%), *CDKN2A* (42%), *SMAD4* (21%), *CDKN2B* (13%), and *FAT3* (13%) (Fig. 3A, left panel), and those in 98 PDAC patients included *KRAS* (94%), *TP53* (75%), *CDKN2A* (36%), and *SMAD4* (27%) (Fig. 3A, right panel). The mutation frequencies in ASCP and PDAC were compared, and the results revealed that the mutational frequencies of *ACVR2A* (6.25% vs 0%, *P* = 0.034), *FANCA* (6.25% vs 0%, *P* = 0.034), *RBM10* (6.25% vs 0%, *P* = 0.034), and *SPTA1* (8.33% vs 1.02%, *P* = 0.04) in ASCP were significantly higher than those in PDAC (Fig. 3B). Importantly, we found a higher rate of 9p21 loss in ASCP than in PDAC (12.5% vs 2.0%, *P* = 0.022) (Fig. 3C). The signalling pathways involving the mutated genes were also compared, and there were obvious differences between enriched pathways in ASCP and PDAC (Fig. S3A, see online supplementary material). The distribution frequency of the Wnt signalling pathway in PDAC was higher than that in ASCP (31.63% vs 27.08%), while the distribution frequency of other signalling pathways, such as HR, cell cycle, and ErbB, in PDAC was lower than that in ASCP. The type and frequencies of *KRAS* mutations were different between the two subtypes, with a significantly higher occurrence of *Q61X* in PDAC (11.2% vs 0%, *P* = 0.03; Fig. S3B). The proportion of samples with low PD-L1 expression in ASCP was higher than that of samples with high PD-L1 expression, while the opposite was true in PDAC, and the difference was not statistically significant (Fig. S3C).

### 9p21 loss may be a new predictor of a poor prognosis in ASCP

**Figure 4.**
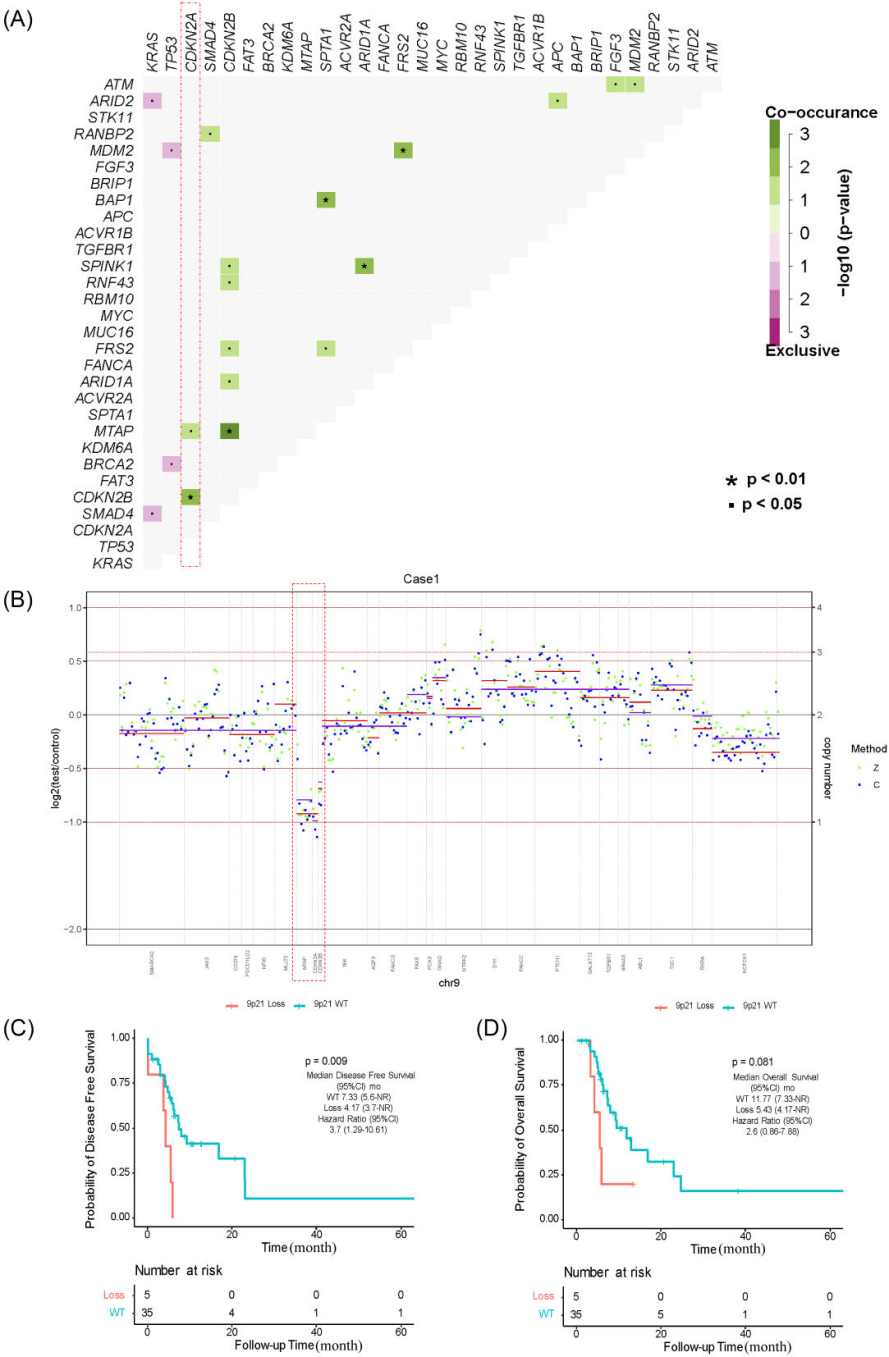
Genetic characteristics and prognosis of 9p21 loss. (**A**) Gene co-mutations in ASCP. (**B**) Example of copy number variation data of chromosome 9. Z and C represent two software algorithms. Genomic region is presented according to the assay used (purple = Z-score, red = C-score) and ordered according to genomic position. Blue spots/purple lines and green spots/red lines indicate the average log-ratio in Z and C, respectively. Lines indicate the average log-ratio in a segment. (**C, D**), Kaplan‒Meier analysis of disease-free survival and overall survival for 9p21, respectively. The hazard ratio was obtained using the Cox proportional hazards test. Loss, homozygous deletion type.

Mutation co-occurrence could provide information for drug combination therapy and medication instruction, therefore gene co-mutation was analysed (Fig. 4A). We obtained 19 pairs of related mutations, including 15 co-occurring mutation pairs and 4 mutually exclusive mutation pairs. *KRAS* mutations and mutations in *ARID2* and *SMAD4* were mutually exclusive, and *TP53* mutations were mutually exclusive with mutations in *MDM2* and *BRAC2*. However, several mutations, such as *ARID1A/SPINK1, CDKN2A/CDKN2B, MTAP/CDKN2B, FRS2*/*MDM2, BAP1*/*SPTA1*, and *CDKN2B/MTAP*, co-occurred to a significant degree. In particular, *MTAP, CDKN2A* and *CDKN2B* were adjacent at the 9p21.3 region, and CNV analysis showed a loss in this region in our cohort (Fig. 2C). We reviewed the original sequencing data from patients who carried 9p21 loss and show the somatic copy number alterations in Fig. 4B and Fig. S4 (see online supplementary material). To further assess the occurrence of 9p21 loss, we performed mutual exclusivity analysis in PAAD from TCGA. Co-deletion of *CDKN2A*/*B* and *MTAP* was frequently observed in 183 patients with CNV (52/183).

To evaluate the prognostic role of 9p21 loss in patients with ASCP, DFS and OS curves were established using Kaplan‒Meier analysis. Among the 6 patients exhibiting 9p21 loss, 4 displayed co-deletion of *CDKN2A*/*CDKN2B*/*MTAP*, while 2 exhibited co-deletion of *CDKN2A*/*CDKN2B*. Complete prognostic information was available for 5 of these patients. Our results revealed that 9p21 loss was significantly related to worse DFS [mDFS, 4.17 vs 7.33 months, 95% confidence interval (CI) 4.17 (3.70-not reached (NR)), *P* = 0.009] (Fig. 4C). The two groups showed similar trends in OS with no significant differences (mOS, 5.43 vs 11.77 months, 95% CI 5.43 (4.17-NR), *P *= 0.081) (Fig. 4D). Survival analysis based on the TCGA-PAAD cohort also found that the 9p21 loss group had a poorer prognosis than the 9p21 WT group (mOS, 16.4 vs 21.7 months, 95% CI 16.4 (14.1–23.1), *P* = 0.039), although there was no significant difference in DFS (Fig. S5A, B, see online supplementary material).

### Prognostic analysis of clinical characteristics

Of the 48 patients investigated during our study, 40 had a complete follow-up with the best objective response assessment as well as DFS and OS evaluations. The correlations between clinical characteristics and prognosis were analysed, revealing that tumour stage, tumour location, and distal metastasis were significantly correlated with DFS (Fig. S6A–C, see online supplementary material). Lymph node metastasis was correlated with DFS, but the correlation did not reach statistical significance (Fig. S6D). Univariate analysis confirmed that distal metastasis was a prognostic factor for DFS and OS (Table S3, see online supplementary material). As observed, the higher the tumour stage and the larger the size, the worse the prognosis. Moreover, surgery was significantly related to DFS and OS (Fig. S6E and F). Thus, the prognosis of patients after radical surgery could be significantly improved.

**Figure 5.**
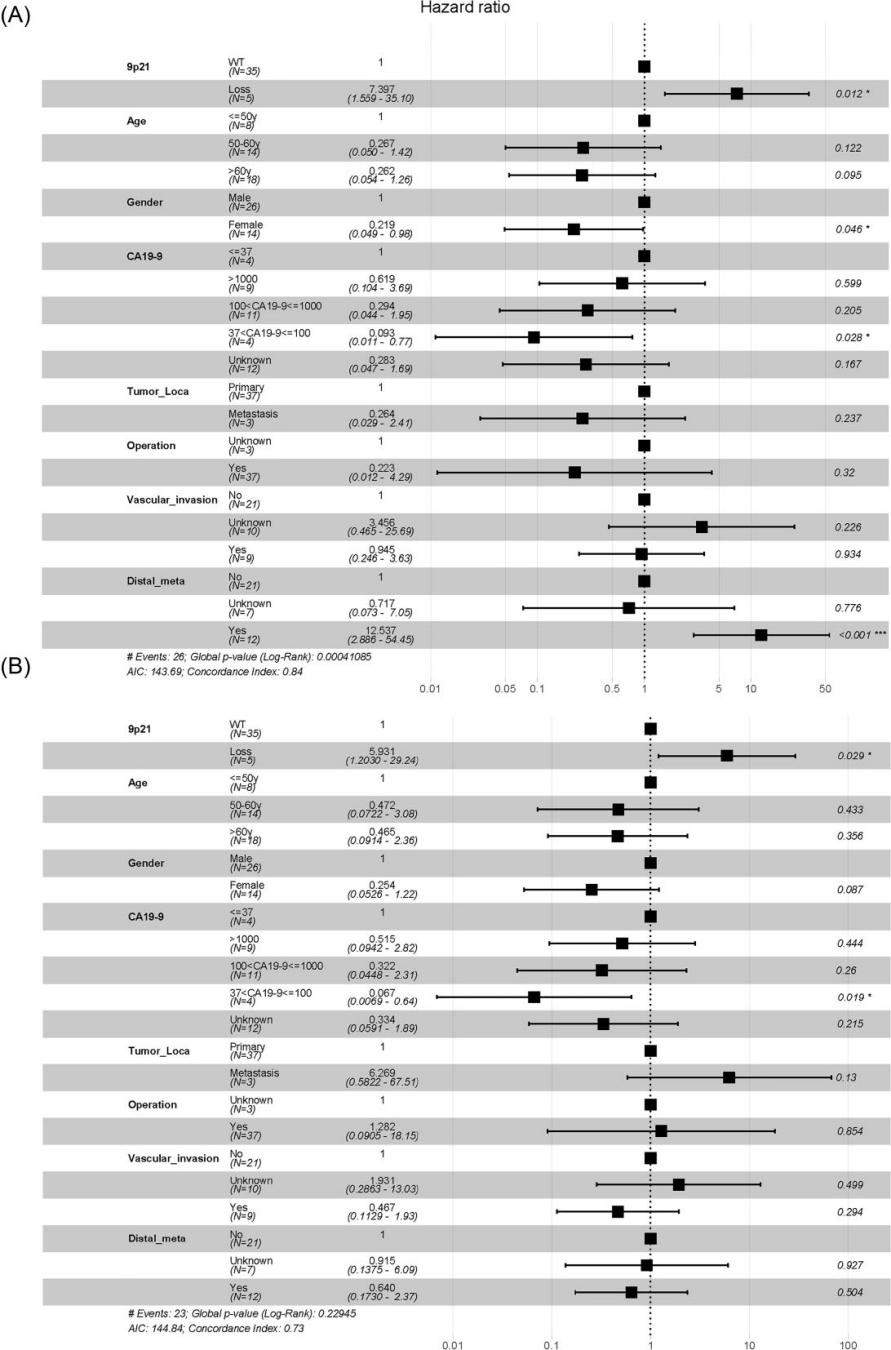
Multivariate analysis of factors associated with DFS and OS. (**A**) Multivariate Cox regression showing that 9p21 (loss/WT) (*P* = 0.012), gender (female/male) (*P *= 0.046), CA19-9 (37 < CA19-9 ≤ 100/≤ 37) (*P* = 0.028), and distal_meta (yes/no) (*P* < 0.001) were significantly associated with DFS. (**B**) Multivariate Cox regression showing that 9p21 (loss/WT) (*P* = 0.029) and CA19-9 (37 < CA19-9 ≤ 100/≤ 37) (*P* = 0.019) might be prognostic indicators of OS in patients with ASCP.

Furthermore, we conducted univariate and multivariate Cox prognostic models for DFS and OS (Table S3 and Fig. 5). Univariate Cox proportional hazard analysis showed that several clinical characteristics were related to survival: these included 9p21 loss, tumour location, operation, tumour size, lymph node metastasis, and distal metastasis (Table S3). Multivariate Cox proportional hazard analysis showed that 9p21 (loss/WT) (*P* = 0.012), gender (female/male) (*P* = 0.046), CA19-9 (37 < CA19-9 ≤ 100/≤ 37) (*P* = 0.028), and distal metastasis (yes/no) (*P* < 0.001) are highly correlated with DFS (Fig. 5A), and 9p21 (loss/WT) (*P* = 0.029) and CA19-9 (37 < CA19-9 ≤ 100/≤ 37) (*P* = 0.019) were associated with OS (Fig. 5B). These results suggest that 9p21 loss could be an independent prognostic factor for OS and DFS.

### 9p21 loss alters gene expression and functional enrichment

We performed differential expression analysis on RNA sequencing data from the TCGA-PAAD cohort. *P* value < 0.05 and |fold change| > 1 were considered the cut-off criteria based on the TCGA cohort. A total of 272 differentially expressed genes (DEGs) were identified, including 179 downregulated genes and 93 upregulated genes (Table S4, see online supplementary material). These results were visualized by a volcano plot (Fig. S7A, see online supplementary material). Importantly, we found that *CDKN2A* and *CDKN2B* were significantly downregulated in the top 30 DEGs in 9p21 loss patients compared to 9p21 WT patients (Fig. S7B). Furthermore, the expression levels of *CDKN2A, CDKN2B*, and *MTAP* were depicted using a violin plot (Fig. S7C). Collectively, these findings provide compelling evidence that 9p21 loss is associated with the downregulation of *CDKN2A* and *CDKN2B* gene expression.

**Figure 6.**
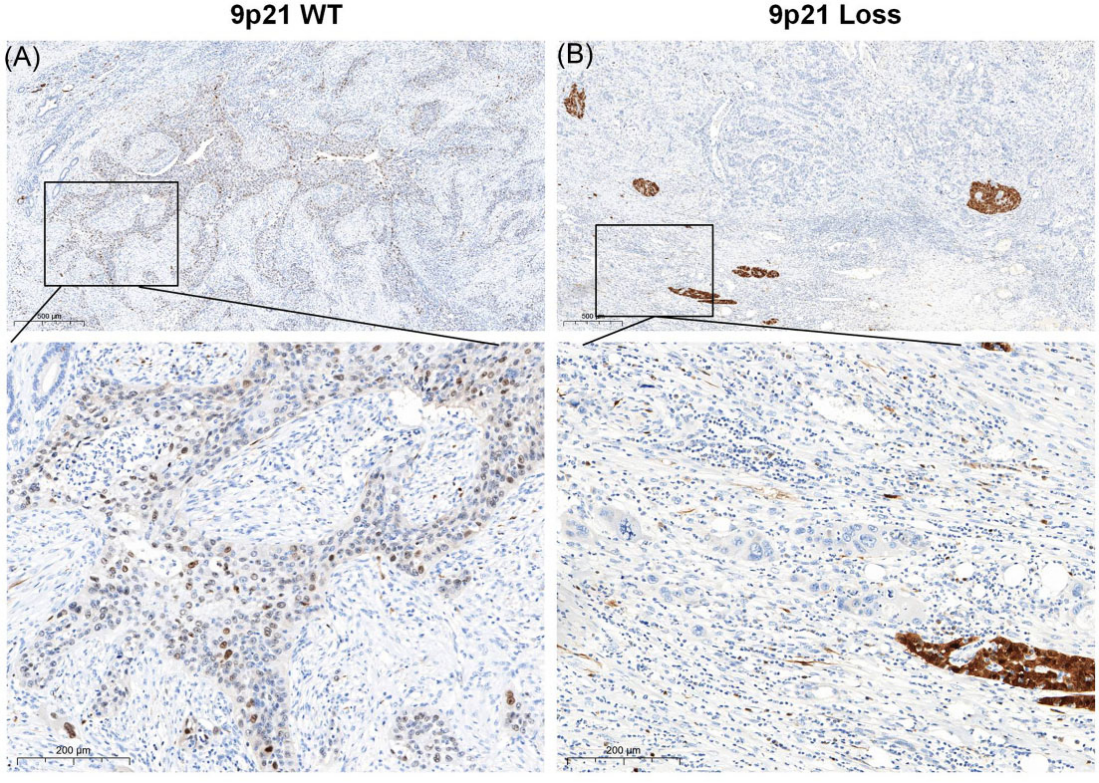
Expression of p16 protein in ASCP specimens. (**A**) IHC for p16-positive status in patients with 9p21 WT. (**B**) IHC for p16-negative status in patients with 9p21 loss.

The *CDKN2A* gene (chromosome 9p21) encodes the protein p16^INK4a^ (also known as p16) through alternative exon usage, and this gene is the second most commonly inactivated tumour suppressor gene in cancer and is lost in the majority of chordomas.^13^ To provide experimental evidence complementing our omics data analysis, we further investigated the protein expression of p16 in tumour tissues with 9p21 loss and 9p21 WT using IHC. Our results showed negative p16 expression in samples harbouring 9p21 loss, while positive p16 expression was observed in 9p21 WT samples (Fig. 6). Furthermore, KEGG analysis showed that the DEGs were involved in several important pathways that promote tumour progression, including the PI3K-Akt signalling pathway, p53 signalling pathway, extracellular matrix (ECM)–receptor interaction, and focal adhesion (Fig. S7D and Table S5, see online supplementary material). This finding suggests that 9p21 loss may be associated with a poor prognosis.

We also observed that 9p21 loss was not strongly associated with TMB in ASCP and PDAC (Fig. S8A, see online supplememtary material). In addition, Han *et al*. performed immune deconvolution analysis of the bulk RNA-seq data from TCGA-PAAD by applying Microenvironment Cell Populations (MCP)-counter.^14^ For PAAD patients with frequent 9p21 loss (vs 9p21-WT tumours), we observed a remarkable decrease in the abundance of T cells, natural killer (NK) cells, myeloid dendritic cells, monocytic lineage, fibroblasts, endothelial cells, cytotoxic lymphocytes, CD4 T cells, and B lineage (Fig. S8B). Moreover, we compared the levels of immune cells between patients with 9p21 WT and patients with 9p21 loss based on ssGSEA algorithms. The results demonstrated that patients with 9p21 WT had significantly higher levels of “activated” dendritic cells (aDCs), B cells, CD8^+^ T cells, cytolytic activity, HLA, inflammation-promoting factors, neutrophils, T cell co-stimulation, Tfh, Th1 cells, and TILs than patients with 9p21 loss (Fig. S8C). GO term enrichment revealed that the DEGs between the two groups were significantly related to the immune response, inflammatory response, and extracellular matrix organization in the BP category; extracellular exosome and plasma membrane in the cellular component (CC) category; and heparin binding and extracellular matrix structural constituent in the MF category (Fig. S9A and Table S5, see online supplementary material). GSEA also showed that immune-related pathways were enriched in patients with 9p21 WT but were not enriched in patients with 9p21 loss (Fig. S9B, C). These results indicate that 9p21 loss promotes the formation of a 'cold' tumor microenvironment (TME) in PAAD. Our results showed a higher rate of 9p21 loss in ASCP than in PDAC, which indirectly suggests that ASCP might be related to the tumour immune microenvironment and a decreased response to immunotherapy.

## Discussion

To the best of our knowledge, this study involved the largest cohort among ASCP genomic landscape studies and prognostic gene variant analyses. Compared with previous studies and the external TCGA-PAAD analyses, we observed a high frequency of *KRAS, TP53, CDKN2A, SMAD4*, and losses of 8q (*MYC*), which is consistent with our report^8^ (Fig. S1). We detected some additional CNVs that have not been reported, such as losses on 7p and 17q and gains on 12q. Lenkiewicz *et al*. detected common recurring PDAC driver events in each of the ASCP genomes, including *CDKN2A* and *SMAD4* homozygous deletions, *KRAS* and *TP53* mutations, and *MYC* amplifications. However, the CNV profiles of each flow-sorted aneuploid population overlapped and included gains of 8q21.3-qtel that contained the MYC locus, a focal amplicon at 15q23-q24.2 that included PKM and CD276, and a homozygous deletion at 5q21.3 targeting EFNA5 and FBXL17.^15^ This indicates that the driver mutation genes of the foreign population and Chinese population are consistent, but there are differences in gene CNV changes. More importantly, we observed a higher proportion of 9p21 loss in ASCP than in PDAC and revealed that 9p21 loss is linked to a poor patient prognosis in ASCP, suggesting that 9p21 loss could function as a prognostic marker in ASCP.

Several published studies have revealed the existence of 9p21 loss in different types of solid tumours, and this loss may have a certain correlation with prognosis. The most prominent homozygous deletions in cancer affect chromosome 9p21.3 and eliminate *CDKN2A/B* tumour suppressors, disabling a cell-intrinsic barrier to tumorigenesis. Barriga *et al*. demonstrated that 9p21.3 deletion not only fails to effectively prevent PDAC cell proliferation but also promotes immune escape while disrupting both intracellular and extracellular tumour suppression programmes.^16^ The results of our study also illustrated for the first time the important prognostic value of 9p21 loss in ASCP, which was consistent with previous studies in other types of tumours. Our findings indicate that 9p21 loss is an adverse prognostic factor for ASCP. 9p21 loss is one of the most frequent somatic copy number alterations that occurs in human cancers.^17^, ^18^ The 9p21.3 region, which includes *MTAP, CDKN2A*, and *CDKN2B*, is pleiotropic, and SNPs in this region spanning the three genes are susceptibility markers for several cancers.^19^ Two crucial tumour suppressor genes located in this region, *CDKN2A* and *CDKN2B*, have well-established roles in cell proliferation and apoptosis and are inactivated in a wide range of cancers.^20^ Additionally, simultaneous deletion mutations for *CDKN2B* and *CDKN2A* are frequently observed in human cancers, including pancreatic cancer.^21^*CDKN2B* deletion is considered essential for pancreatic cancer development instead of co-deletion with *CDKN2A*.^22^ Biallelic deletions of *MTAP* with the neighbouring *CDKN2A* are commonly observed in 40% of pancreatic cancers.^23^ Therefore, the prognostic factor for ASCP should be validated in larger sample sizes and polyethnic studies. In the present study, we found that all *CDKN2B* deletions occurred within the adjacent domains that included *CDKN2A* or *CDKN2A/MTAP*. The real mutant forms included losses in the 9p21.3 region that were verified with actual sequencing data, which were significantly associated with DFS, indicating the important prognostic role of 9p21 loss in ASCP. Apart from their prognostic value, anti-tumour agents are also the focus of clinical research. In our study, we analysed the DEGs between patients with 9p21 loss and patients with 9p21 WT and found that the expression of *CDKN2A* and *CDKN2B* was significantly upregulated in patients with 9p21 WT compared to patients with 9p21 loss. These results indicate that 9p21 loss leads to the downregulation of *CDKN2A* and *CDKN2B* gene expression. The two gene products of the *CDKN2A* gene, p16 and p19ARF, have recently been linked to each of two major tumour suppressor pathways in human carcinogenesis, the RB1 pathway and the p53 pathway.^24^ These results suggest that 9p21 loss results in *CDKN2A* and *CDKN2B* gene expression and then promotes tumour progression. Of course, this is also a limitation of our study, and we will further investigate its mechanism and potential biological behaviour through basic experiments in future studies.

Furthermore, as precision medicine advances, the characterization of the genomic landscape will directly influence clinical therapeutic decision making. Patients with targetable alterations receiving matching targeted therapy survive 1 year longer than those receiving standard therapies.^25^ In our ASCP cohort, 48% of patients harboured potential actionable alterations, including *CDKN2A/B, PIK3CA, NF1, FBXW7, STK11, ATM, BRCA2, FGFR1, PTEN*, and *KRAS* mutations. As mentioned above, CDK inhibitors could be a therapeutic option for patients with *CDKN2A/CDKN2B* mutations. Patients with a BRCA2 mutation reportedly respond well to single-agent poly (ADP-ribose) polymerase (PARP) inhibitors.^26^ The US Food and Drug Administration has approved the application of PARP inhibitors, such as olaparib, for germline BRCA-mutated pancreatic cancers.^27^ Patients with *ATM* mutations, which play an important role in the HR pathway, may also benefit from PARP inhibitors. Additionally, *PI3K, AKT*, and mammalian target of rapamycin (m*TOR*) inhibitors may serve as potential therapeutic options for patients with *PIK3CA* and *PTEN* mutations, whereas an *mTOR* inhibitor can be used as a therapeutic approach for targeting *STK11-* and *FBXW7*-deficient tumours.^27–29^*NF1* inactivation leads to the activation of the mTOR pathway and promotes tumour cell growth; therefore, mTOR inhibitors can be used in potential therapies for *NF1*-deficient patients.^30^*KRAS* is the most common oncogene in pancreatic cancers, and several inhibitors targeted to *KRAS* G12C have been tested in clinical trials, including adagrasib and sotorasib, which have shown some evidence of efficacy.^31^ The high proportion of patients with actionable alterations in our ASCP cohort could provide reliable evidence for targeted therapy and improve the outcomes of patients with ASCP.

In addition to targeted therapy, immunotherapy is an important treatment method for patients with tumours. TMB is a predictive biomarker in cases of high values, indicating a high rate of response to immune checkpoint inhibitors (ICIs).^32^ Studies have also verified that immunotherapy shows promising results for PDAC patients with high TMB.^33^ However, TMB in ASCP is not yet fully understood. In our study, the TMB status in the 48 patients with ASCP was assessed: only one patient (2.1%) was defined as TMB-H, a similar proportion to that among PDAC patients.^34^, ^35^ PD-L1 expression is another important biomarker of ICIs. In previous studies, ∼11% of ASCPs were positive for PD-L1, with a 10% cut-off value for PD-L1 positivity^25^. In our cohort, 12.5% (4/32) of patients had high PD-L1 expression with a cut-off of CPS ≥ 10. Furthermore, we explored whether mutated genes and altered signalling pathways can influence the TMB values in ASCP. We found that *ACVR2A, BRCA2, CASP8, CDKN2A, EPHB1, FRS2*, and *SPTA1* mutations led to a significantly higher TMB, whereas *KRAS* and *STK11* mutations led to significantly lower TMB. Several studies have proposed that 9p21 loss may be related to the failure of immunotherapy and result in worse outcomes.^14^, ^36^ In our study, 9p21 loss seemed to be more common in ASCP than in PDAC, which indirectly suggests that ASCP has a worse prognosis and more adverse immune reactivity. In the TCGA-PAAD cohort, GSEA showed that immune-related pathways were enriched in patients with 9p21 WT but were not enriched in patients with 9p21 loss. In addition, patients with 9p21 WT had significantly higher levels of aDCs, B cells, CD8^+^ T cells, cytolytic activity, HLA, inflammation-promoting factors, neutrophils, T-cell co-stimulation, Tfh, Th1 cells, and tumour-infiltrating lymphocyte (TIL) than patients with 9p21 loss. These findings indicated that tumours with 9p21 loss showed a cold tumour-like immunophenotype, which may be related to the suppression of immune cell recruitment, the promotion of T-cell activation and immunoregulatory factor expression, and the upregulation of immunosuppressive signals, which jointly induce the cold immunophenotype of tumours with 9p21 deletion. More immunotherapy data need to be gathered in pancreatic cancer to evaluate the influence of 9p21 loss in the future.

To investigate the molecular differences between ASCP and PDAC, mutational characteristics were analysed, and the frequencies of mutated genes between ASCP and PDAC were compared. Our results demonstrated that the genomic characteristics in ASCP and PDAC had some similarities, but there were also differences. The mutational frequencies of *ACVR2A, FANCA, RBM10*, and *SPTA1* in ASCP were significantly higher than those in PDAC. *ACVR2A, FANCA*, and *RBM10* mutations were detected only in patients with ASCP. It seemed that *ACVR2A, FANCA, RBM10*, and *SPTA1* mutations might have important functions in tumour development for different subtypes and different molecular characteristics in Chinese patients. We also observed a higher frequency of *Q61X* in PDAC than in ASCP, which means that the molecular mechanism of *KRAS* mutations may be different between the two subtypes. A larger cohort is needed for further verification.

ASCP has a poor prognosis, and there is limited data to predict prognosis and to guide optimal treatment. Therefore, we examined the associations between clinical characteristics and prognosis in all respects. The screening and identification of new prognostic biomarkers in patients with pancreatic cancer, especially those with ASCP, is urgently needed for clinical decision-making. Tumour location, CA19-9 level, and tumour size have been identified as preoperative, independent, predictive risk factors for poor prognosis in patients with PDAC.^37^ A retrospective study revealed that for patients with advanced pancreatic cancer, tumour stage, chemotherapy, circulating regulatory T cells, CA19-9 levels, CA125 levels, and *KRAS* G12D and G12V mutations are significantly associated with OS.^38^ Lymph node metastasis appears to correlate with a poor prognosis in patients with pancreatic adenocarcinoma, and surgical resection has a better prognosis than nonsurgical resection for patients with resectable pancreatic cancer.^39^, ^40^ The results of univariate Cox regression analyses in our study also showed that surgery was significantly associated with DFS and OS. Although there was no statistical significance, our data also showed that lymph node metastasis tended to be associated with an adverse prognosis. Therefore, early and accurate diagnosis is crucial for the outcome of patients after radical surgery. In addition, tumour stage and distal metastasis were significantly correlated with DFS, but OS was not significant. One possible reason is that the follow-up time was not long enough to gather reliable data for the long-term response. Interestingly, the results of univariate Cox regression analyses and multivariate analysis both showed that 9p21 loss was related to poor prognosis and could function as an independent risk factor for poor prognosis in patients with ASCP. An increasing number of studies have suggested that 9p21 loss is related to poor survival.^14^, ^41^, ^42^ Overall, prompt intervention and detection of patients with ASCP, and especially attention and measures for patients with 9p21 loss, are needed to prolong life expectancy.

In summary, we comprehensively investigated the genomic variations in ASCP and explored the specific mutation patterns of ASCP and prognostic-related molecular markers, providing a molecular basis for differential diagnosis, prognosis prediction, and the exploration of new treatment options for ASCP. However, limited information is available for targeted and immunological therapy in ASCP. We look forward to obtaining more treatment data for the benefit of patients and to further exploring the tumour immune microenvironment to predict immunotherapy responses for patients. Our results displayed a higher rate of 9p21 loss in ASCP than in PDAC. Notably, 9p21 loss was significantly associated with a poor prognosis in ASCP patients. The results of our study also illustrated for the first time the important prognostic value of 9p21 loss in ASCP and that ASCP might have a more adverse immune reactivity than PDAC.

## Supplementary Material

pbad030_Supplemental_Files
