# Supplementary material for: Loss of chromosome 9p21 is associated with a poor prognosis in adenosquamous carcinoma of the pancreas
Source: Precis Clin Med. 2023 Nov 7;6(4):pbad030. doi: 10.1093/pcmedi/pbad030 (PMC10681361; doi:10.1093/pcmedi/pbad030)
Supplement: pbad030_Supplemental_Files [file pbad030_supplemental_files.zip › Supplementary figure legends.docx]

# Supplementary figure legends

**Supplementary Figure 1.** Mutation frequencies were compared between our sequencing data and data of TCGA database. The mutation frequency was calculated based on the intersection of the YuanSu™ 450 gene panel.

**Supplementary Figure 2.** Correlation between TMB and gene alterations and functional enrichment analysis in 48 ASCP patients. (A) Mutation of genes associated with TMB vs. wild type. (B) KEGG pathway analysis indicated pathway enrichment, and GO analysis indicated the gene functions for mutated genes. (C-D) Mutated signalling pathways significantly associated with TMB. MT, mutated type; WT, wild type; TMB, tumour mutational burden.

**Supplementary Figure 3.** Gene mutation and PD-L1 expression were compared in the ASCP and PDAC groups. (A) Comparison of the signalling pathway mutations between ASCP and PDAC. (B) Comparison of *KRAS* mutations. (C) Comparison of PD-L1 expression. MT, mutated type; BP, biological process; CC, cellular component; MF, molecular function; WT, wild type.

**Supplementary Figure 4**. Copy number variation data of chromosome 9 for five cases.

**Supplementary Figure 5**. Kaplan‒Meier survival curves comparing the loss and wild type of 9p21 in pancreatic adenocarcinoma (PAAD) based on the TCGA database. (A–B) Survival curves of DFS and OS in the PAAD cohort (n = 183).

**Supplementary Figure 6**. Relationship between clinical characteristics and survival in ASCP. The hazard ratio was obtained from the Cox proportional hazard test. (A–D), Kaplan‒Meier analysis of disease-free survival (DFS) for tumour location, distal metastasis, stage, and lymph node status. (E) and (F), Kaplan‒Meier analysis of DFS and overall survival for surgical operation.

**Supplementary Figure 7.** Differential Gene Expression and Functional Analysis Associated with 9p21 Loss. (A) The volcano map shows the DEGs between the 9p21 loss and 9p21 WT groups. (B) The heatmap shows the top 30 DEGs. (C) Violin plot displaying the distribution of gene expression in the different groups. (D) KEGG pathway analysis of DEGs.

**Supplementary Figure 8**. The relationship between 9p21 loss and TMB in ASCP and PDAC and the association of 9p21 loss with immune cells. (A) The difference in TMB (9p21-Loss vs. 9p21-WT) between ASCP and PDAC. (B) The role of 9p21 loss in shaping the immune cell abundance and cell composition in the tumour microenvironment. Immune deconvolution was performed by applying MCP-counter to the bulk RNA-seq data. The bar plot was drawn using computed log2-transformed fold change (9p21-Loss vs. 9p21-WT) and adjusted p values (FDR q-value). (C) Levels of immune cells were compared between patients with 9p21 loss and 9p21 WT. P value < 0.05 is marked as *, p value < 0.01 is marked as **, and p value < 0.001 is marked as ***.

**Supplementary Figure 9.** Functional analysis of differentially expressed genes associated with 9p21 loss. (A) GO analysis. (B) GSEA of patients with 9p21 WT. (C) GSEA of patients with 9p21 loss.
