# Supplementary material for: Loss of chromosome 9p21 is associated with a poor prognosis in adenosquamous carcinoma of the pancreas
Source: Precis Clin Med. 2023 Nov 7;6(4):pbad030. doi: 10.1093/pcmedi/pbad030 (PMC10681361; doi:10.1093/pcmedi/pbad030)
Supplement: pbad030_Supplemental_Files [file pbad030_supplemental_files.zip › Table S3.docx]

Table S3 Univariate Cox regression analyses for risk score of patients with ASCP.

| **Variable** | **DFS** | | ***P* value** | **OS** | | ***P* value** |
| --- | --- | --- | --- | --- | --- | --- |
|  | **HR** | **95% CI** |  | **HR** | **95% CI** |  |
| Model cohort (n = 40) | | | | | | |
| **9p21** |  |  |  |  |  |  |
| WT | Reference | |  | Reference | |  |
| Loss | 3.703 | 1.292-10.612 | **0.015** | 2.604 | 0.86-7.884 | 0.091 |
| **Age** |  |  |  |  |  |  |
| ≤ 50 | Reference | |  | Reference | |  |
| 50-60 | 0.582 | 0.199-1.708 | 0.325 | 0.766 | 0.225-2.605 | 0.669 |
| > 60 | 0.587 | 0.215-1.605 | 0.299 | 0.756 | 0.235-2.428 | 0.638 |
| **Gender** |  |  |  |  |  |  |
| Male | Reference | |  | Reference | |  |
| Female | 0.837 | 0.377-1.861 | 0.663 | 0.708 | 0.303-1.657 | 0.426 |
| **Tumor differentiation** |  |  |  |  |  |  |
| Low | Reference | |  | Reference | |  |
| M_Low | 1.291 | 0.499-3.341 | 0.599 | 1.113 | 0.396-3.129 | 0.839 |
| Unknown | 2.788 | 0.831-9.357 | 0.097 | 2.526 | 0.72-8.854 | 0.148 |
| **CA19-9** |  |  |  |  |  |  |
| ≤ 37 | Reference | |  | Reference | |  |
| 37 < CA19-9 ≤ 100 | 0.479 | 0.077-2.964 | 0.429 | 0.368 | 0.058-2.32 | 0.287 |
| 100 < CA19-9 ≤ 1000 | 1.061 | 0.267-4.219 | 0.933 | 0.713 | 0.173-2.94 | 0.639 |
| > 1000 | 1.406 | 0.361-5.476 | 0.623 | 0.72 | 0.178-2.914 | 0.645 |
| Unknown | 1.02 | 0.255-4.077 | 0.978 | 0.509 | 0.116-2.223 | 0.369 |
| **Tumor location** |  |  |  |  |  |  |
| Primary | Reference | |  | Reference | |  |
| Metastasis | 3.831 | 1.115-13.166 | **0.033** | 6.073 | 1.668-22.103 | **0.006** |
| **Operation** |  |  |  |  |  |  |
| Unknown | Reference | |  | Reference | |  |
| Yes | 0.014 | 0.001-0.145 | **<0.001** | 0.178 | 0.037-0.858 | **0.031** |
| **R0** |  |  |  |  |  |  |
| Other | Reference | |  | Reference | |  |
| R0 | 0.541 | 0.23-1.272 | 0.159 | 0.420 | 0.167-1.059 | 0.066 |
| **Tumor size** |  |  |  |  |  |  |
| T1 | Reference | |  | Reference | |  |
| T2 | 1.635 | 0.196-13.666 | 0.650 | 1.652 | 0.198-13.816 | 0.643 |
| T3 | 2.933 | 0.375-22.965 | 0.305 | 1.941 | 0.245-15.4 | 0.53 |
| T4 | 2.125 | 0.23-19.637 | 0.506 | 2.041 | 0.224-18.641 | 0.527 |
| Unknown | 157.216 | 7.59-3256.681 | **0.001** | 10.157 | 0.829-124.529 | 0.07 |
| **Lymph node metastasis** |  |  |  |  |  |  |
| No | Reference | |  | Reference | |  |
| Yes | 2.162 | 0.886-5.278 | 0.090 | 1.576 | 0.66-3.759 | 0.305 |
| Unknown | 97.446 | 9.118-1041.476 | **<0.001** | 6.701 | 1.327-33.85 | **0.021** |
| **Vascular invasion** |  |  |  |  |  |  |
| No | Reference | |  | Reference | |  |
| Yes | 1.21 | 0.455-3.216 | 0.702 | 0.996 | 0.349-2.844 | 0.994 |
| Unknown | 1.863 | 0.734-4.728 | 0.190 | 2.114 | 0.775-5.769 | 0.144 |
| **Nerve invasion** |  |  |  |  |  |  |
| No | Reference | |  | Reference | |  |
| Yes | 0.905 | 0.348-2.357 | 0.839 | 0.629 | 0.23-1.716 | 0.365 |
| Unknown | 1.701 | 0.655-4.42 | 0.275 | 1.774 | 0.644-4.882 | 0.267 |
| **Distal metastasis** |  |  |  |  |  |  |
| No | Reference | |  | Reference | |  |
| Yes | 4.304 | 1.868-9.92 | **<0.001** | 1.038 | 0.419-2.571 | 0.936 |
| Unknown | 0.57 | 0.127-2.555 | 0.463 | 0.832 | 0.233-2.976 | 0.777 |
