# Supplementary figures and images for: Loss of chromosome 9p21 is associated with a poor prognosis in adenosquamous carcinoma of the pancreas
Source: Precis Clin Med. 2023 Nov 7;6(4):pbad030. doi: 10.1093/pcmedi/pbad030 (PMC10681361; doi:10.1093/pcmedi/pbad030)

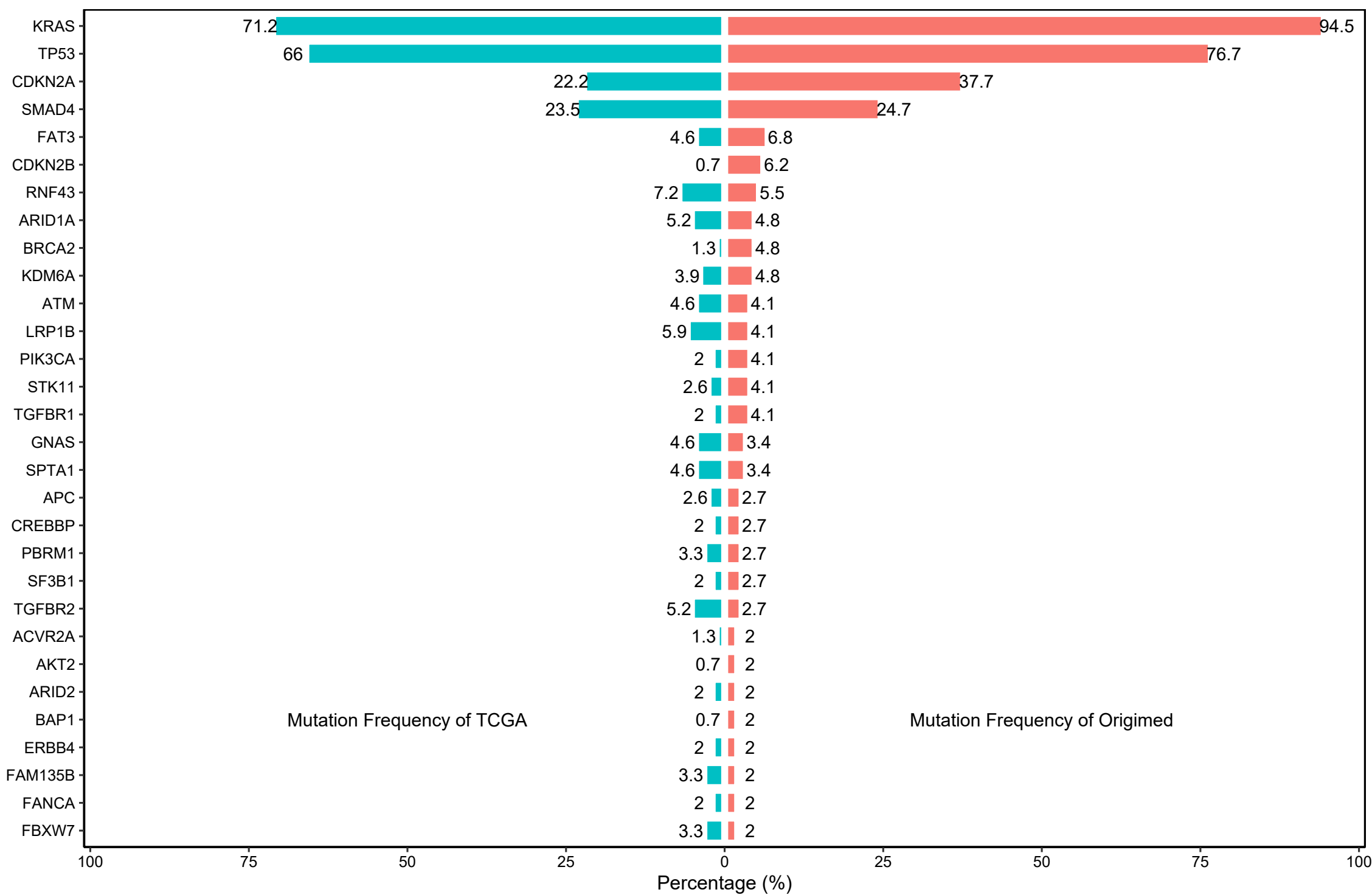

Supplement: pbad030_Supplemental_Files [file pbad030_supplemental_files.zip › Fig. S1.pdf]

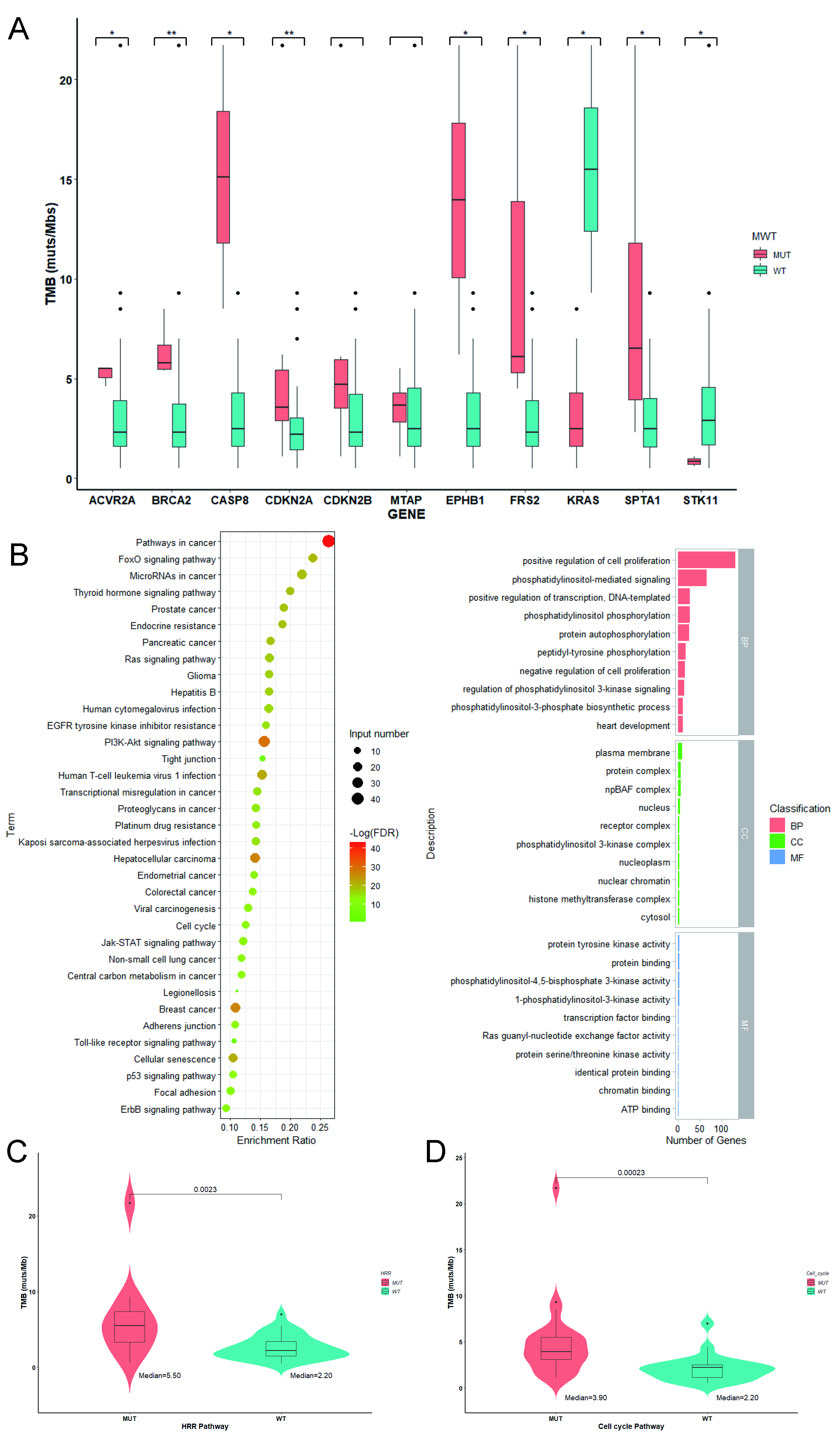

Supplement: pbad030_Supplemental_Files [file pbad030_supplemental_files.zip › Fig. S2.tif]

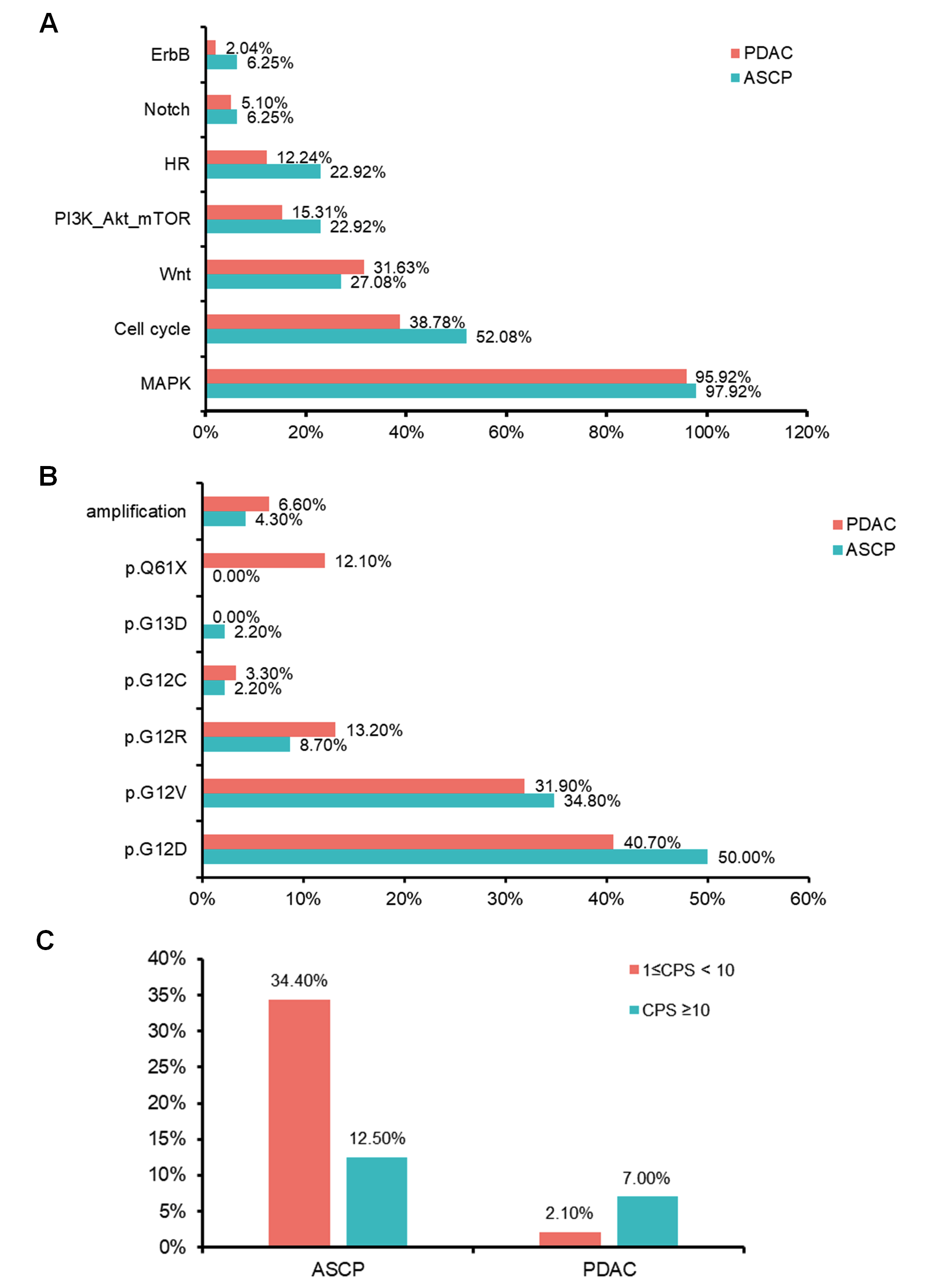

Supplement: pbad030_Supplemental_Files [file pbad030_supplemental_files.zip › Fig. S3.tiff]

Case 2

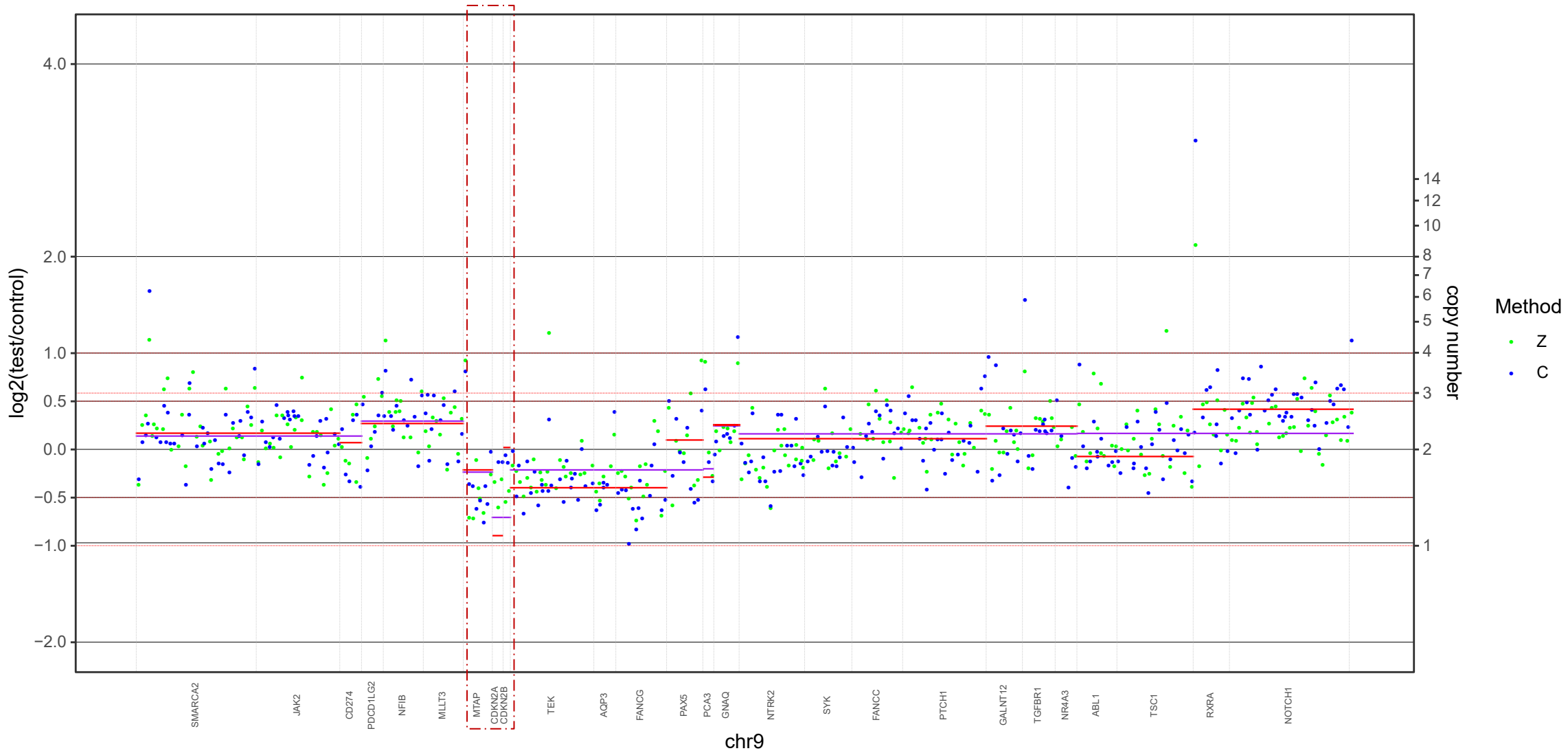

Case 3

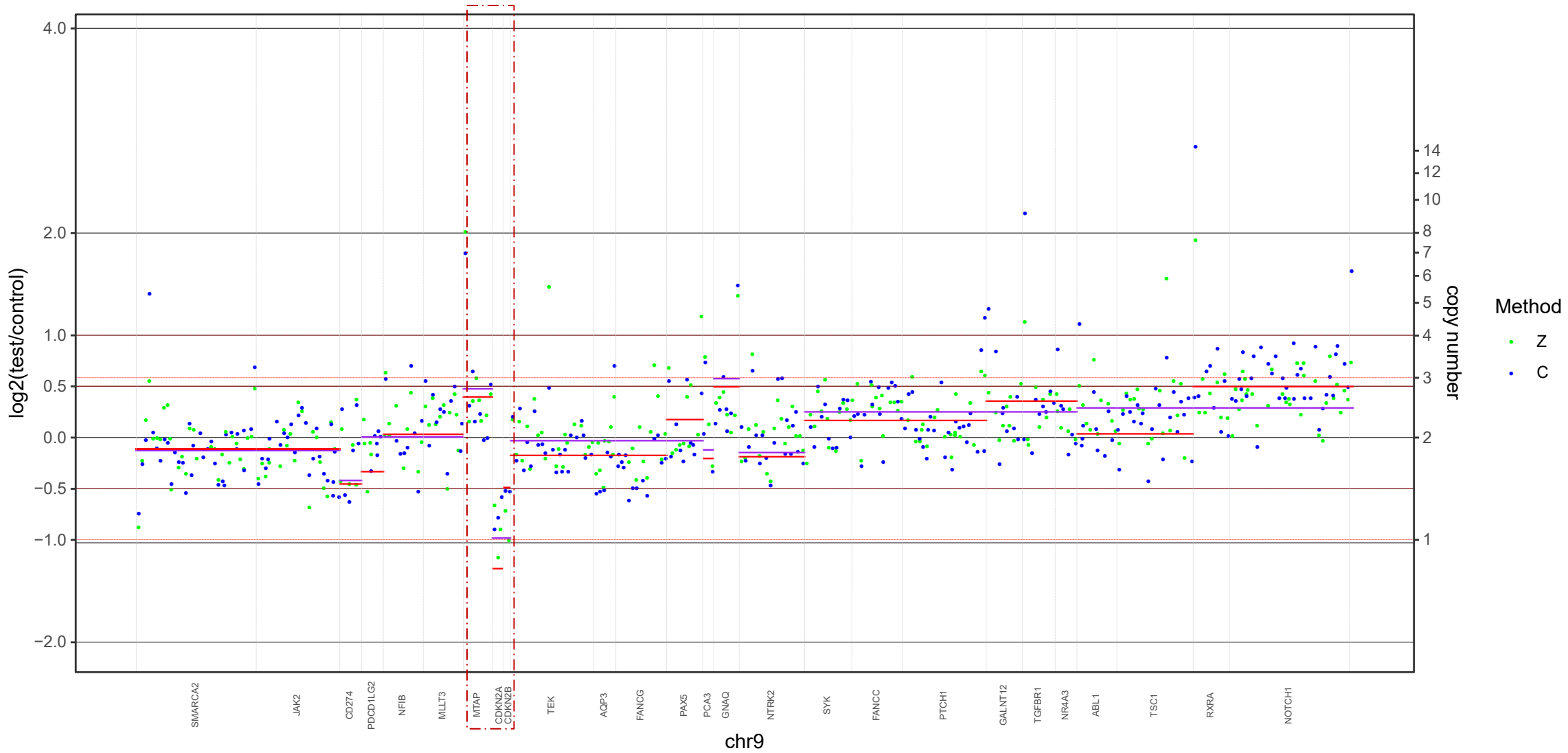

Case 4

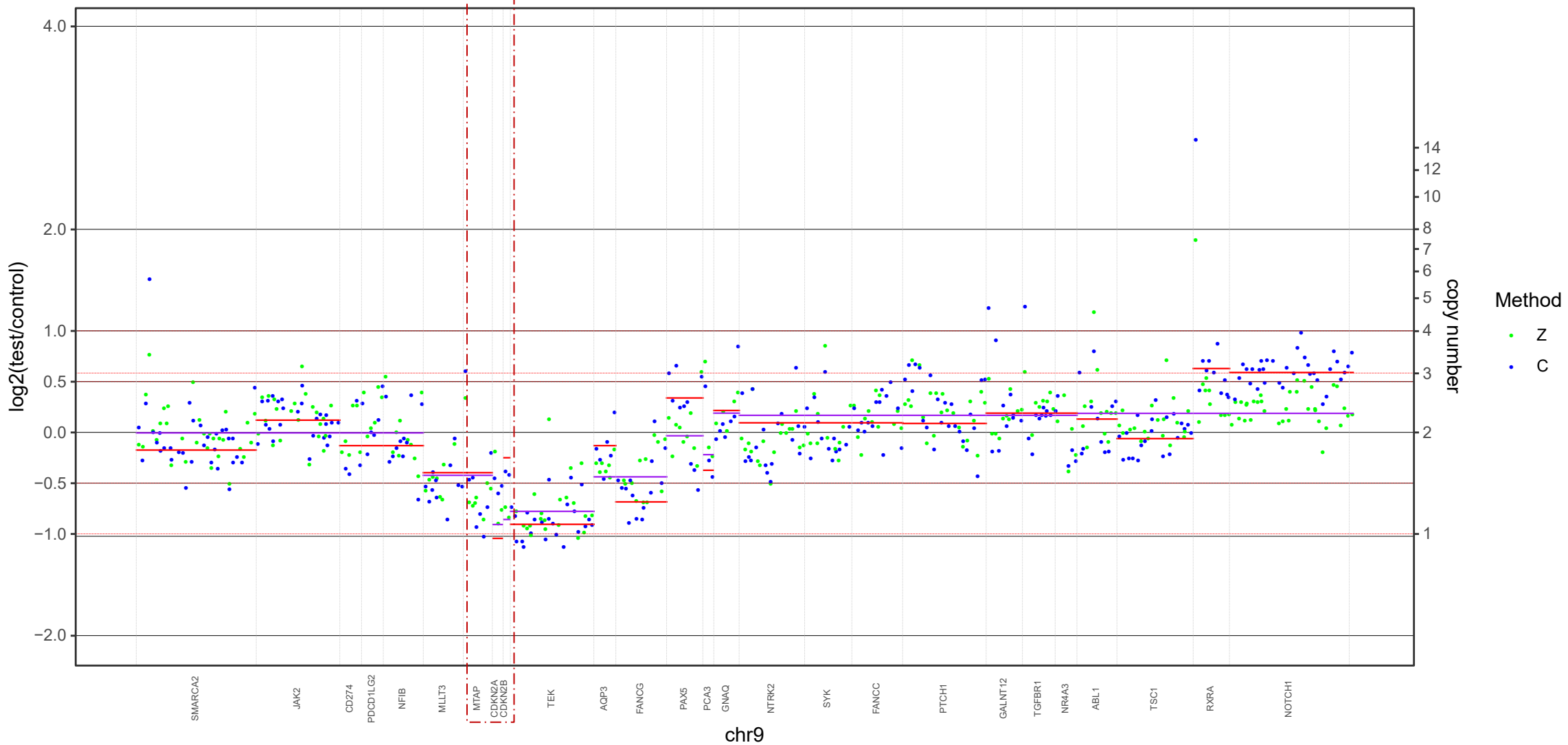

Case 5

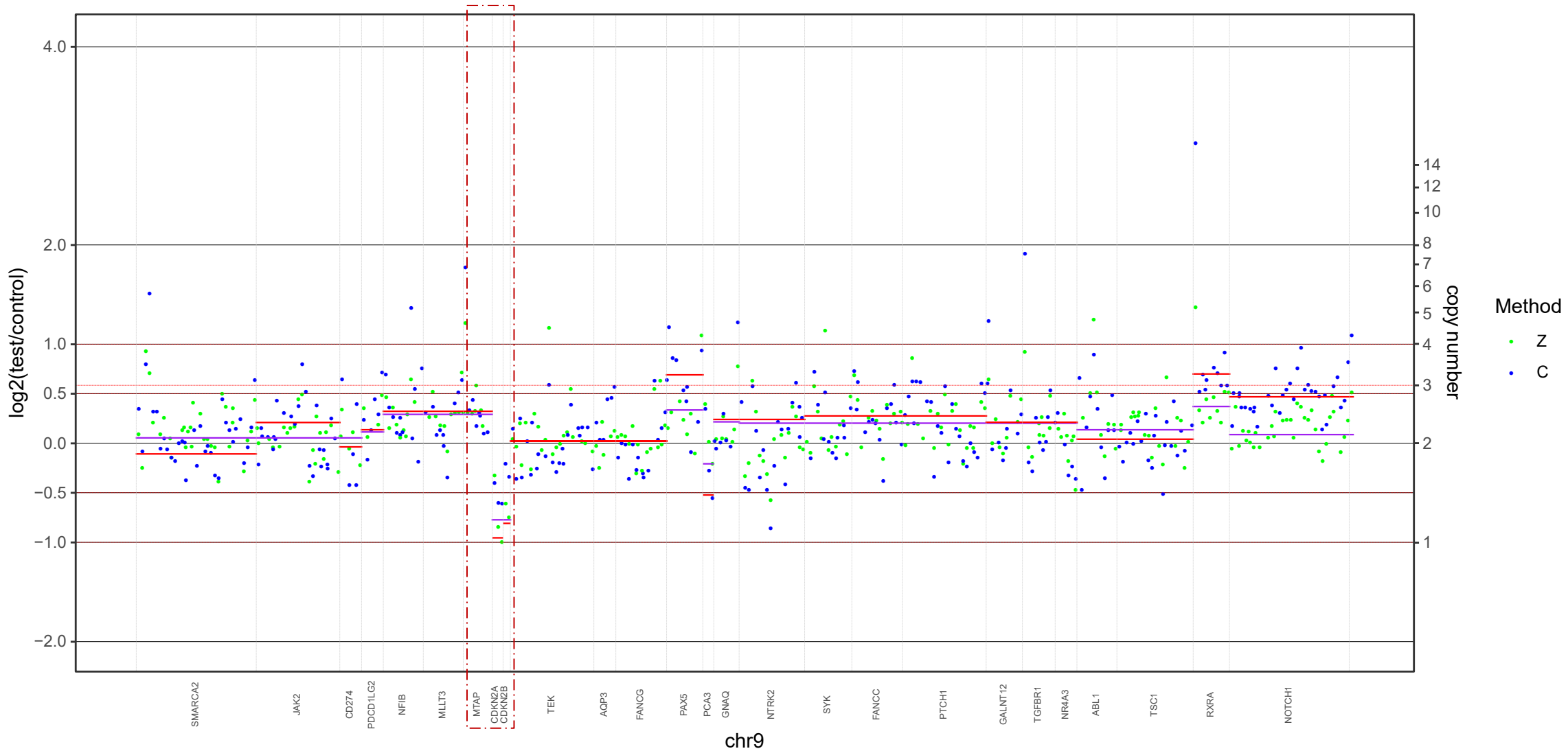

Case 6

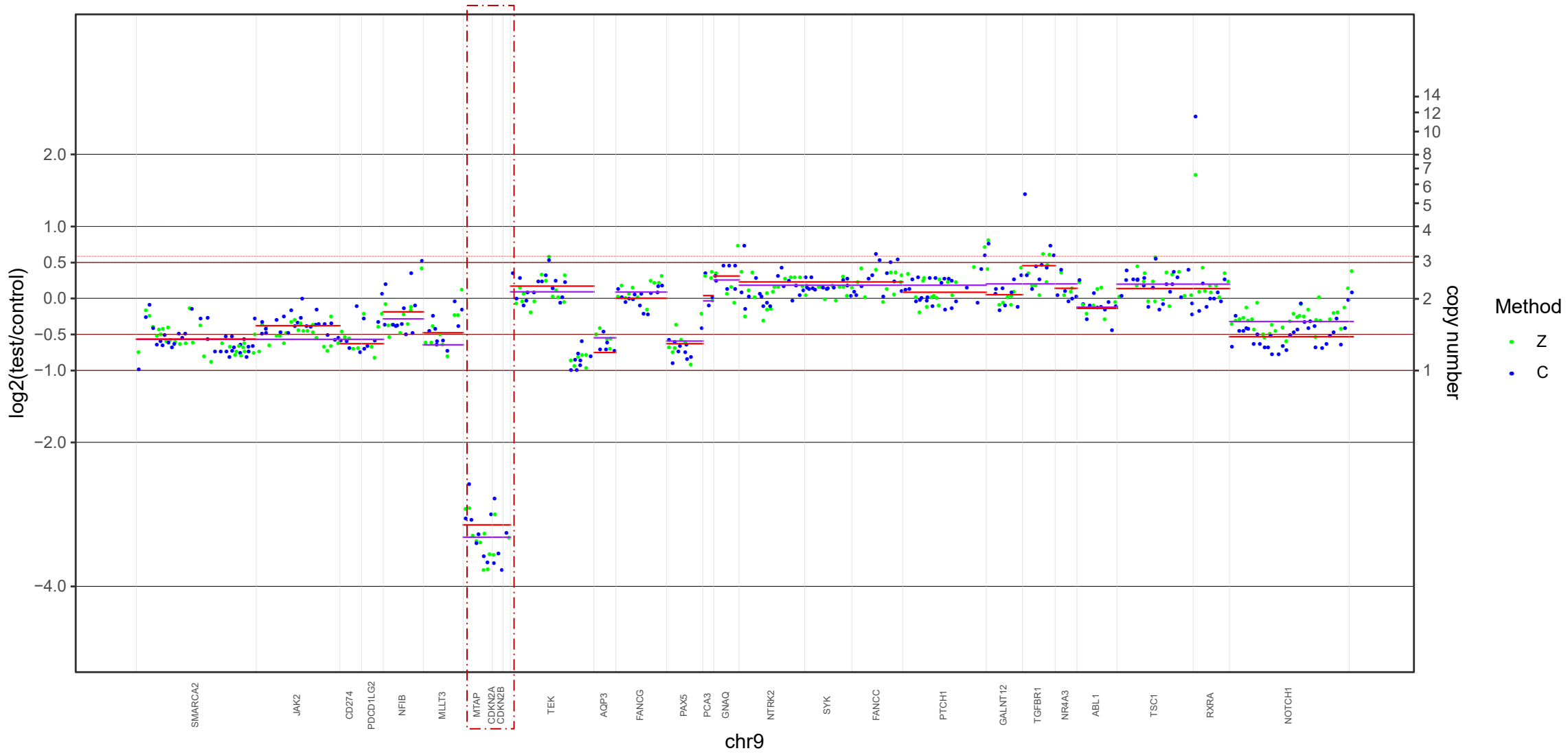

Supplement: pbad030_Supplemental_Files [file pbad030_supplemental_files.zip › Fig. S4.pdf]

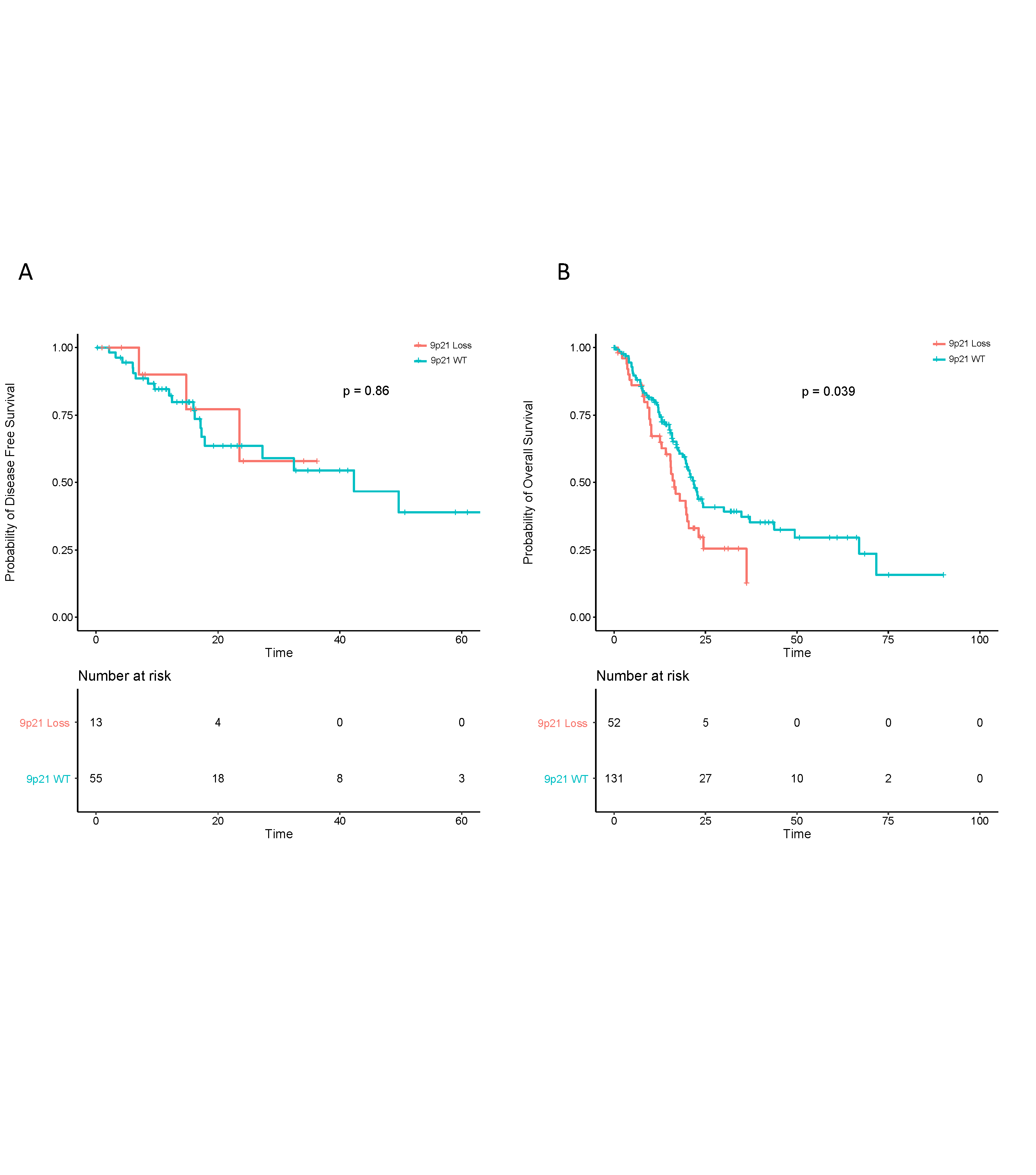

Supplement: pbad030_Supplemental_Files [file pbad030_supplemental_files.zip › Fig. S5.tiff]

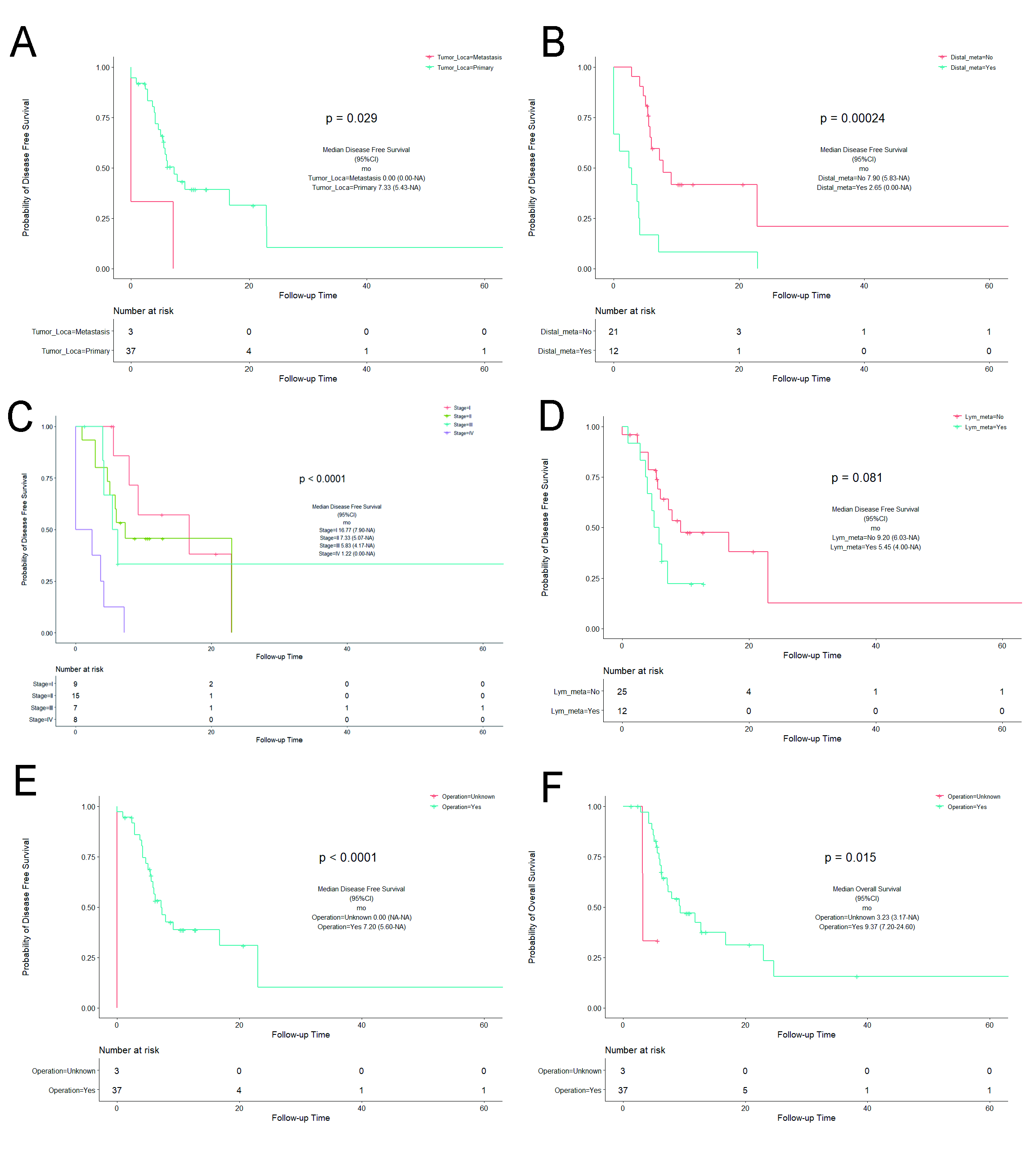

Supplement: pbad030_Supplemental_Files [file pbad030_supplemental_files.zip › Fig. S6.tif]

A

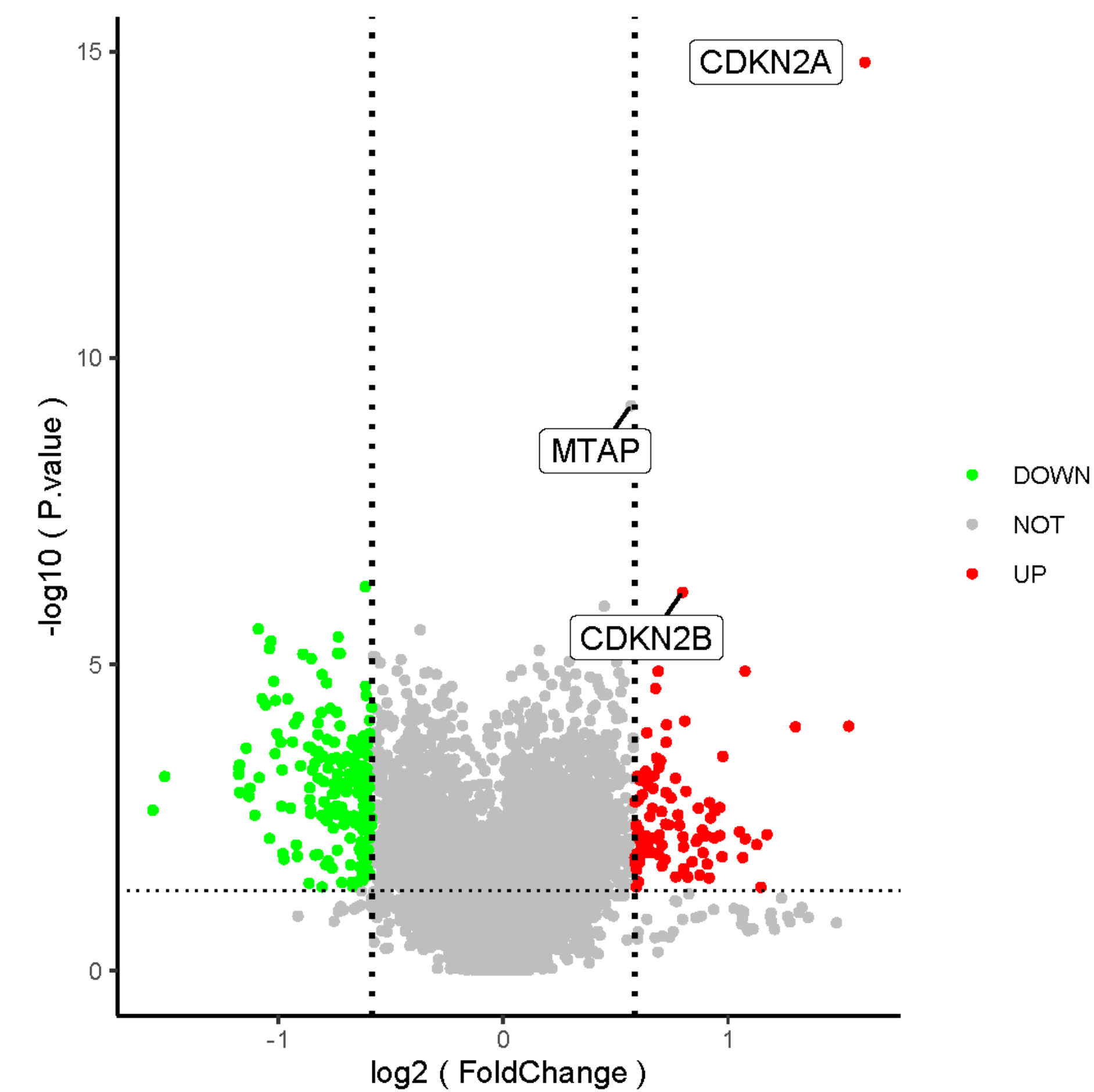

B

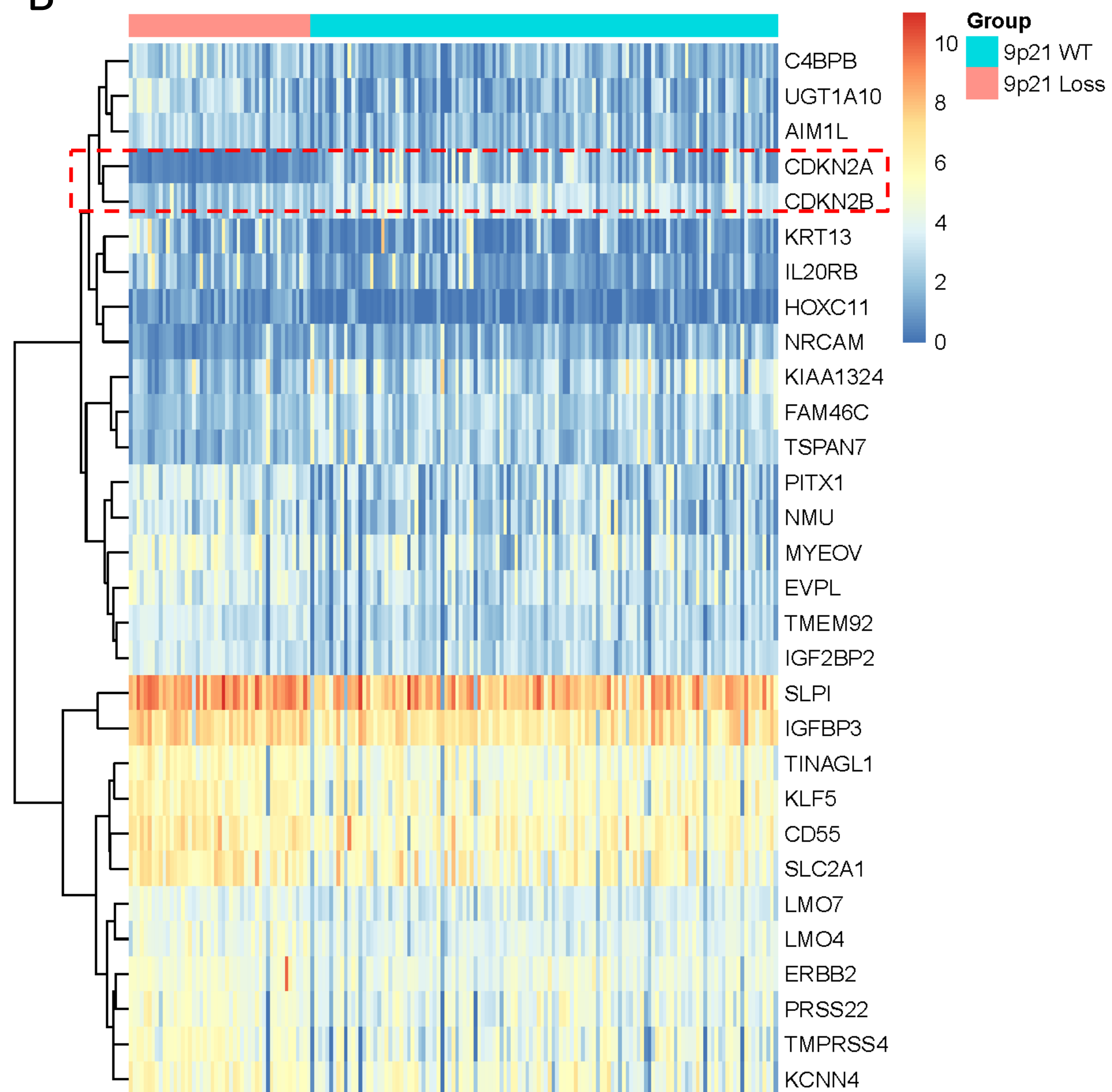

C

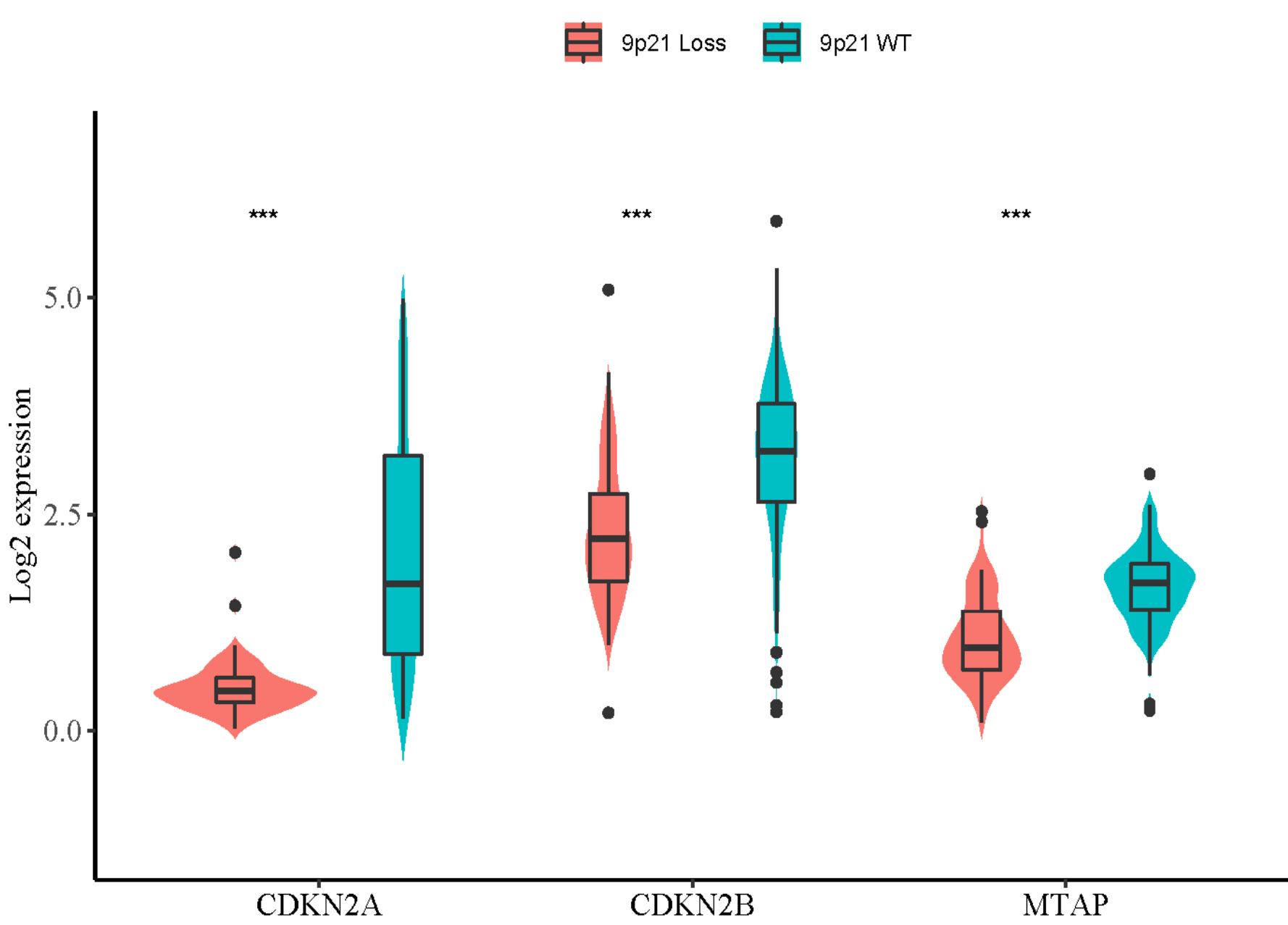

D

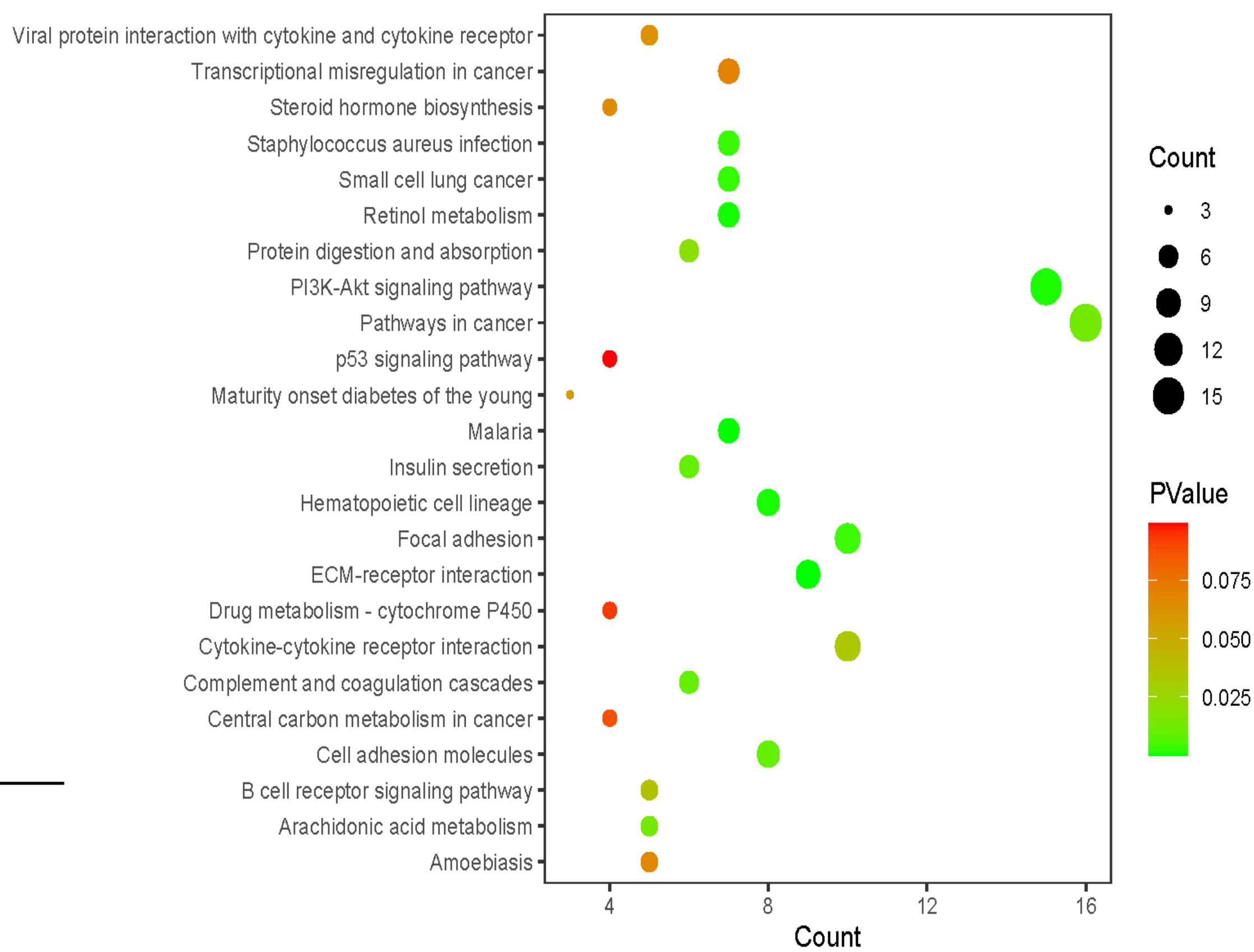

Supplement: pbad030_Supplemental_Files [file pbad030_supplemental_files.zip › Fig. S7.pdf]

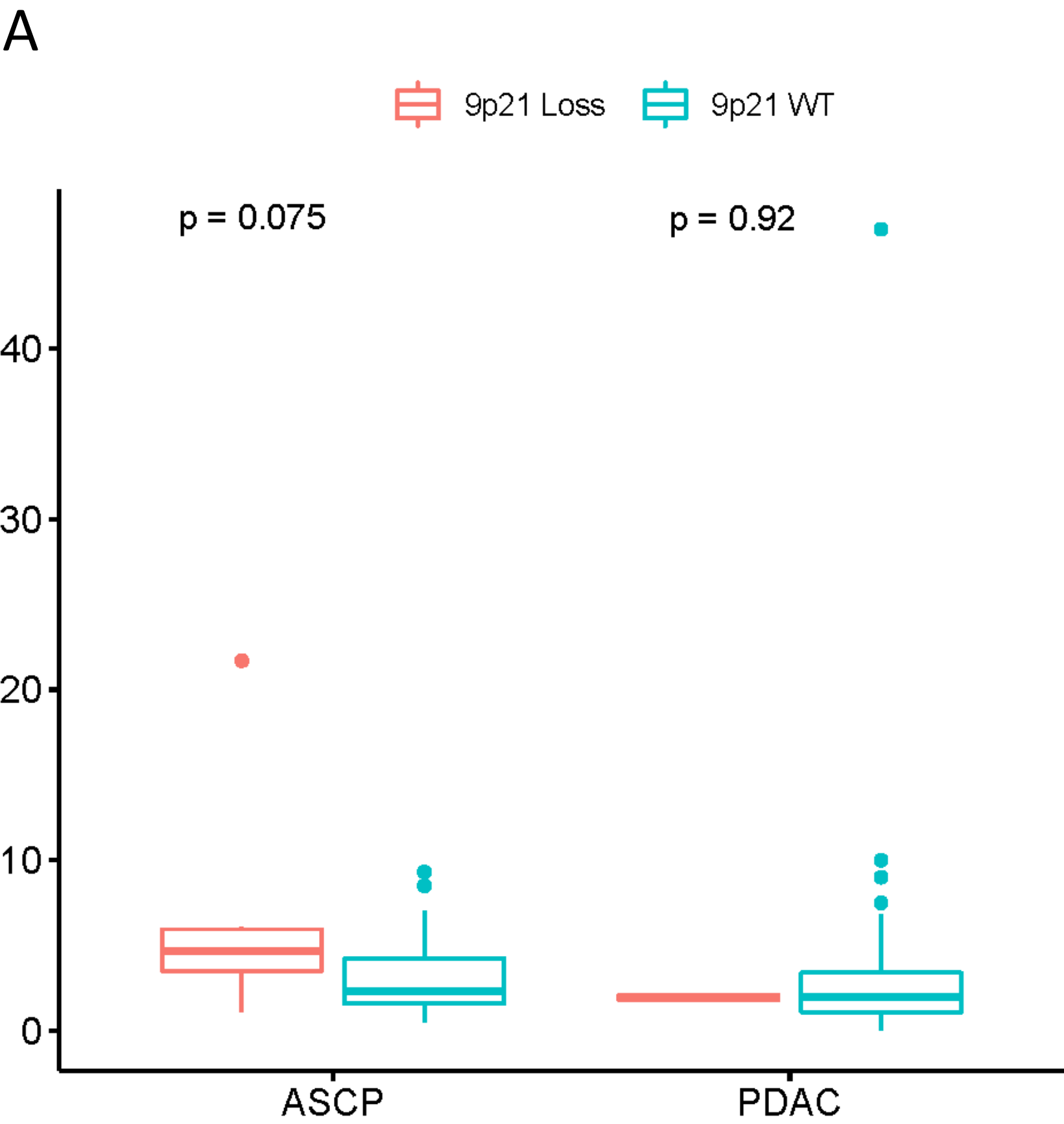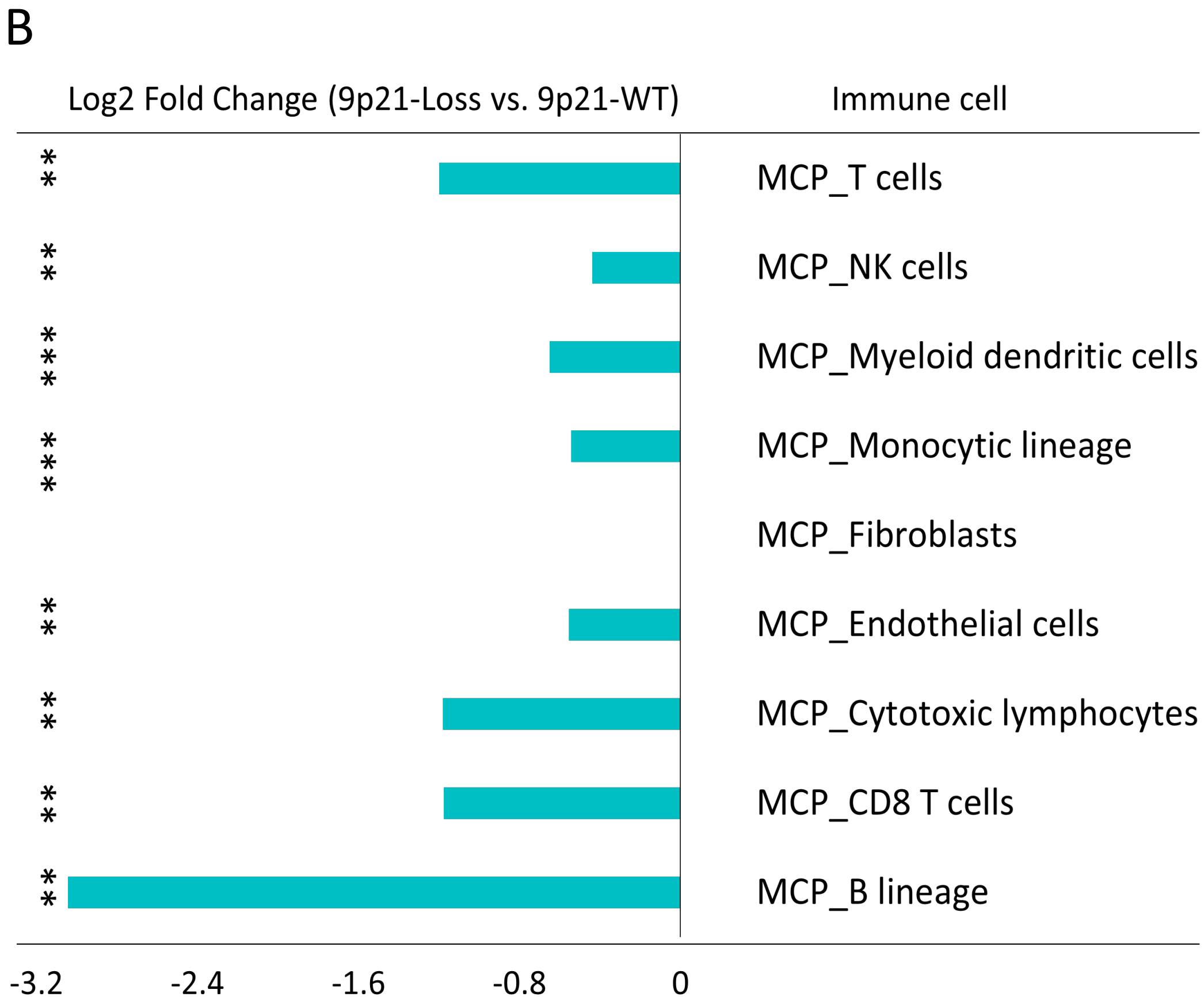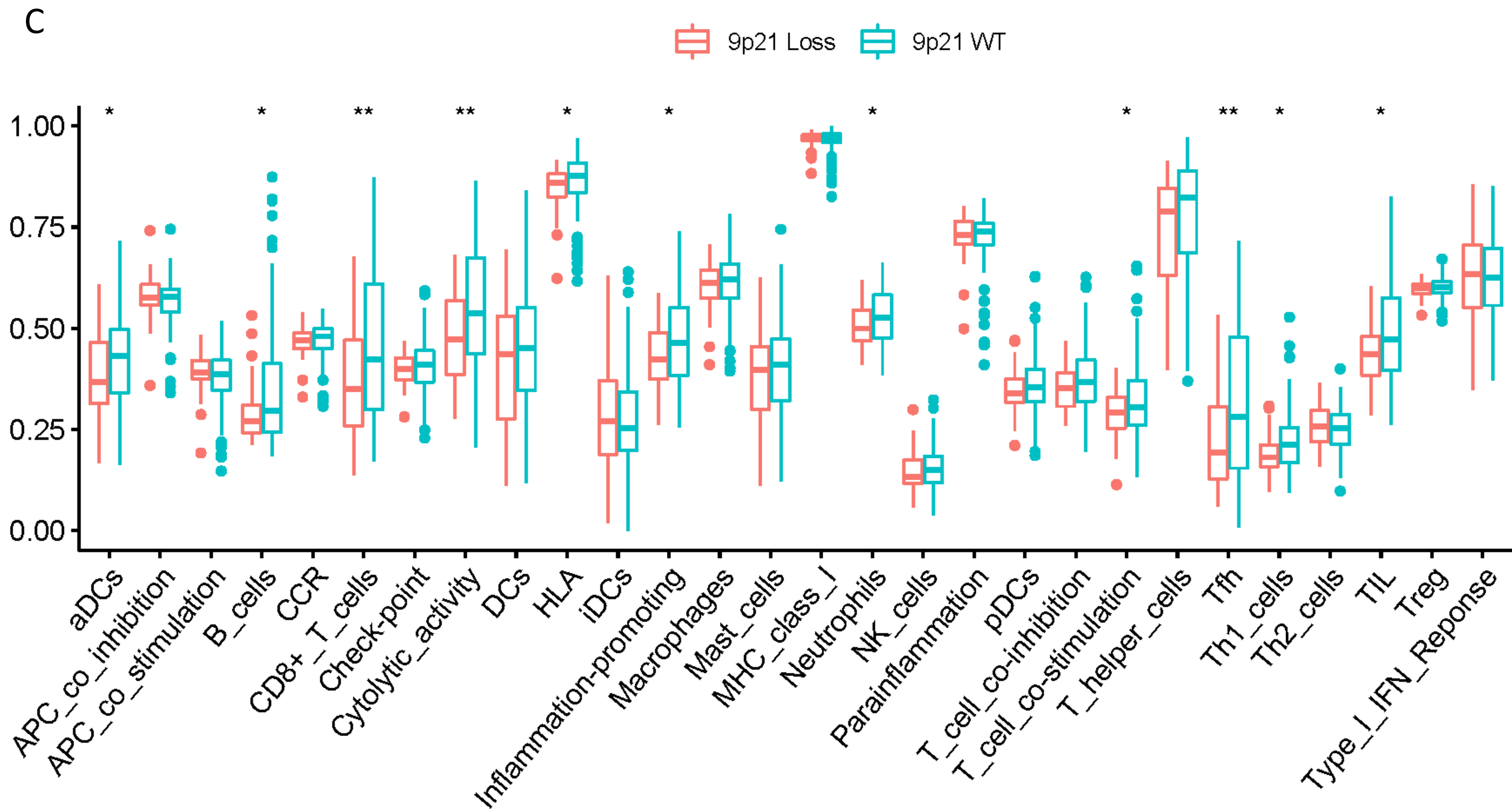

Supplement: pbad030_Supplemental_Files [file pbad030_supplemental_files.zip › Fig. S8.pdf]

A

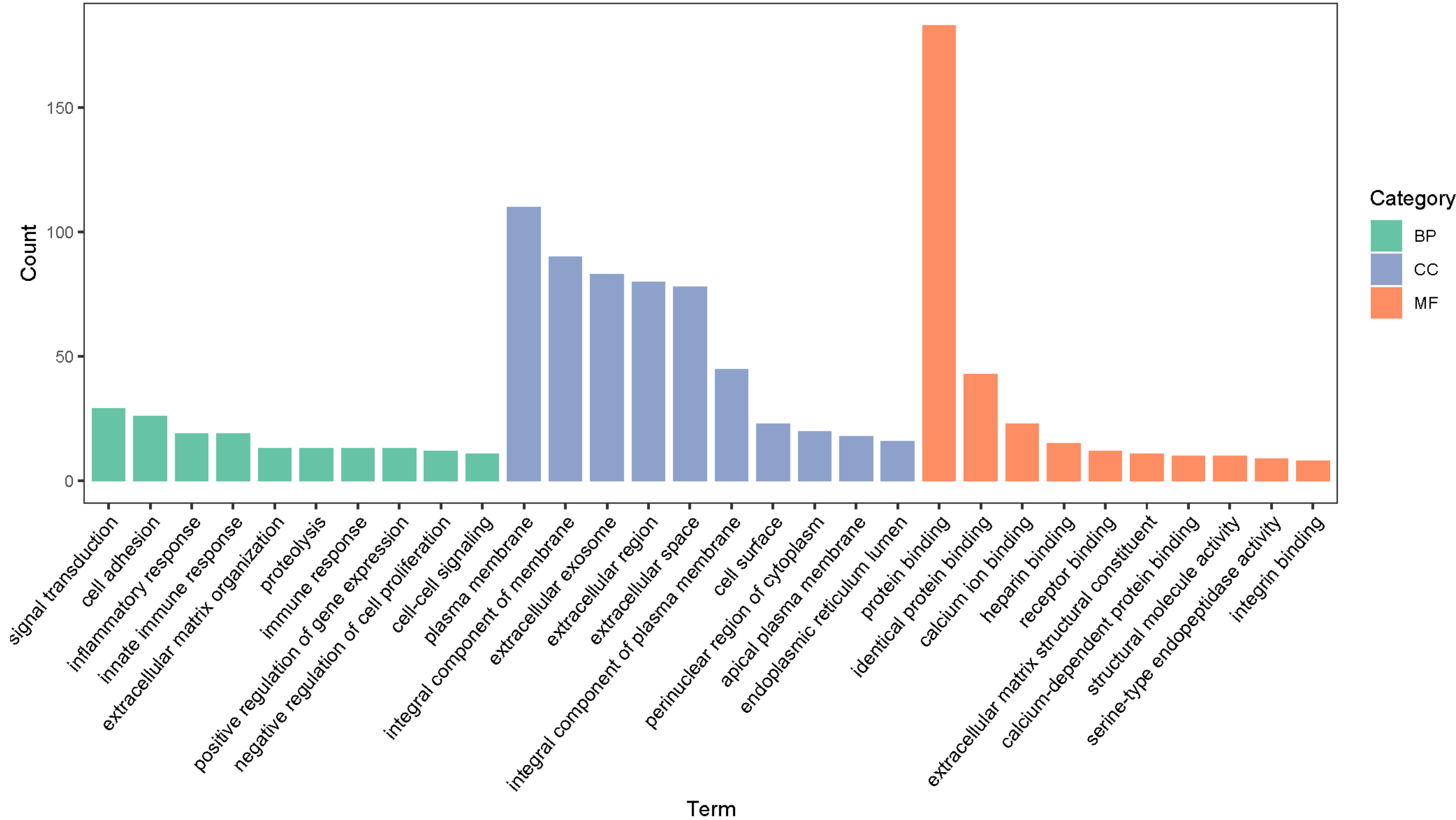

B

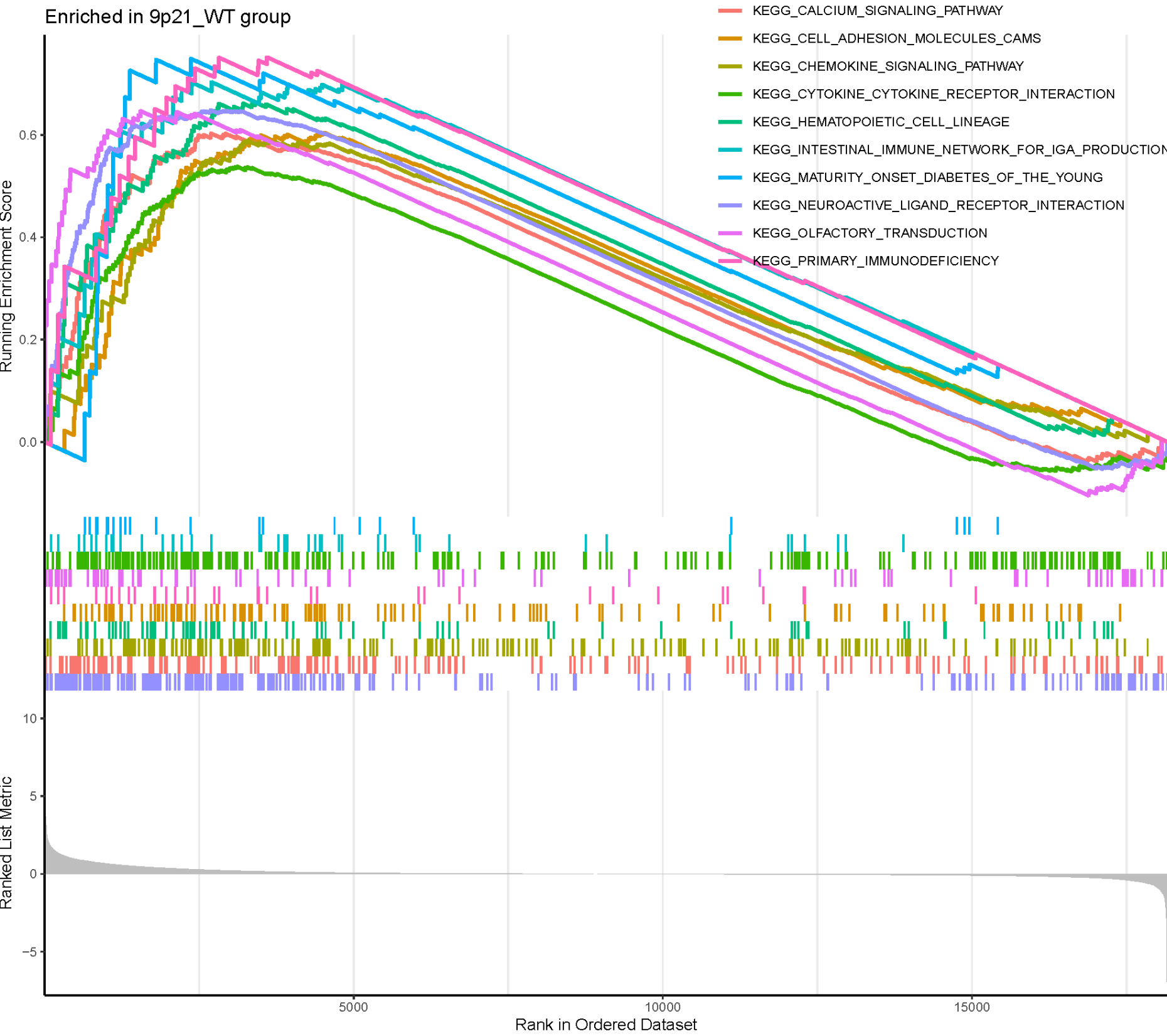

C

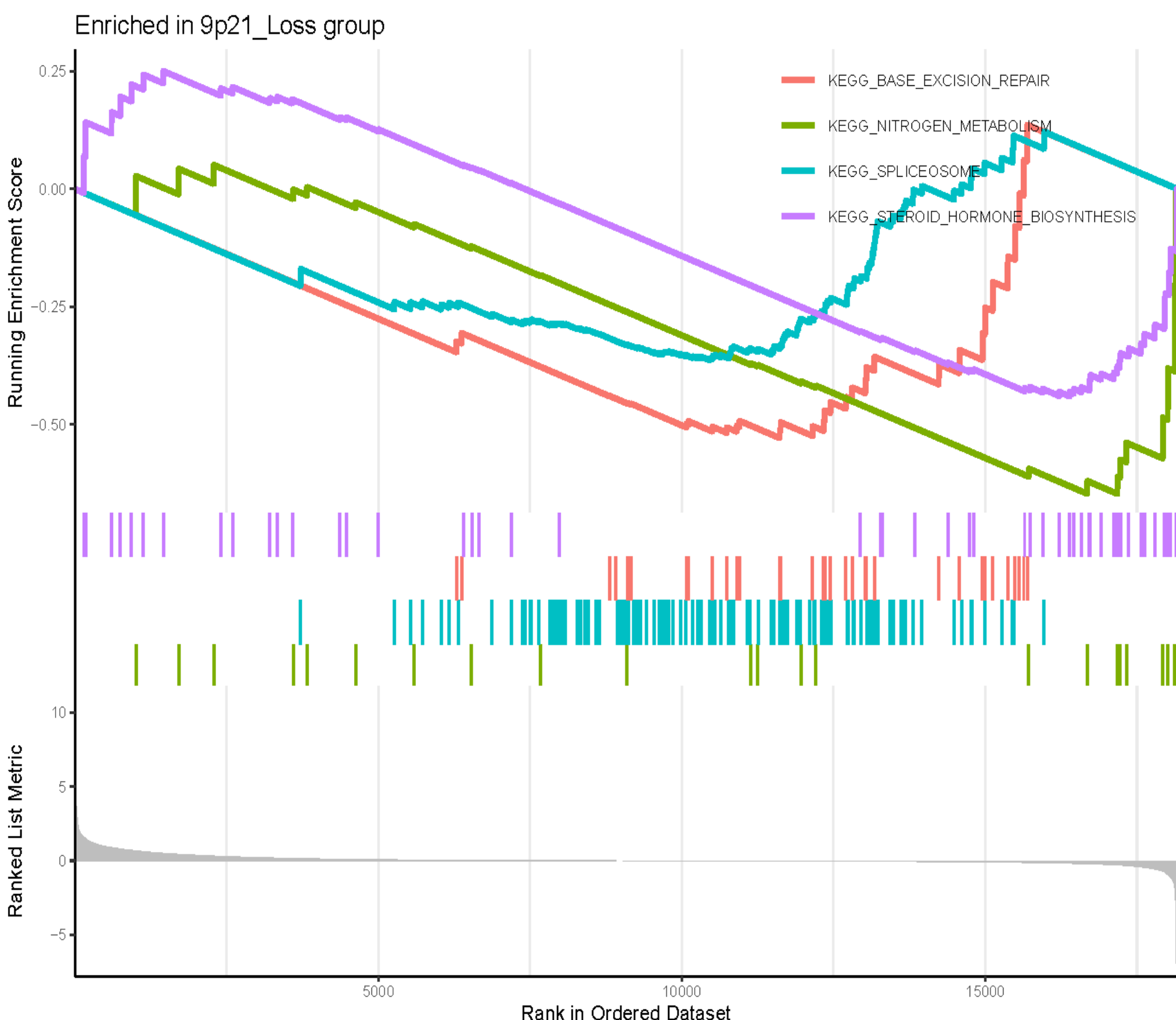

Supplement: pbad030_Supplemental_Files [file pbad030_supplemental_files.zip › Fig. S9.pdf]
